# Defects in ER–endosome contacts impact lysosome function in hereditary spastic paraplegia

**DOI:** 10.1083/jcb.201609033

**Published:** 2017-05-01

**Authors:** Rachel Allison, James R. Edgar, Guy Pearson, Tania Rizo, Timothy Newton, Sven Günther, Fiamma Berner, Jennifer Hague, James W. Connell, Jürgen Winkler, Jennifer Lippincott-Schwartz, Christian Beetz, Beate Winner, Evan Reid

**Affiliations:** 1 Cambridge Institute for Medical Research, University of Cambridge, Cambridge CB2 0XY, England, UK; 2 Department of Medical Genetics, University of Cambridge, Cambridge CB2 0XY, England, UK; 3 Department of Clinical Biochemistry, University of Cambridge, Cambridge CB2 0XY, England, UK; 4 Interdisciplinary Center for Clinical Research (IZKF) Junior Research Group III and Federal Ministry of Education and Research (BMBF) Research Group Neuroscience, Friedrich-Alexander-University Erlangen-Nuernberg, 91054 Erlangen, Germany; 5 Department of Molecular Neurology, Friedrich-Alexander-University Erlangen-Nuernberg, 91054 Erlangen, Germany; 6 Institute of Human Genetics, Friedrich-Alexander-University Erlangen-Nuernberg, 91054 Erlangen, Germany; 7 Department of Clinical Chemistry and Laboratory Diagnostics, Jena University Hospital, 07743 Jena, Germany; 8 Janelia Research Campus, Howard Hughes Medical Institute, Ashburn, VA 20147

## Abstract

Contacts between endosomes and the endoplasmic reticulum (ER) promote endosomal tubule fission, but the mechanisms involved and consequences of tubule fission failure are incompletely understood. We found that interaction between the microtubule-severing enzyme spastin and the ESCRT protein IST1 at ER–endosome contacts drives endosomal tubule fission. Failure of fission caused defective sorting of mannose 6-phosphate receptor, with consequently disrupted lysosomal enzyme trafficking and abnormal lysosomal morphology, including in mouse primary neurons and human stem cell–derived neurons. Consistent with a role for ER-mediated endosomal tubule fission in lysosome function, similar lysosomal abnormalities were seen in cellular models lacking the WASH complex component strumpellin or the ER morphogen REEP1. Mutations in *spastin*, *strumpellin*, or *REEP1* cause hereditary spastic paraplegia (HSP), a disease characterized by axonal degeneration. Our results implicate failure of the ER–endosome contact process in axonopathy and suggest that coupling of ER-mediated endosomal tubule fission to lysosome function links different classes of HSP proteins, previously considered functionally distinct, into a unifying pathway for axonal degeneration.

## Abstract (precis)

Hereditary spastic paraplegia (HSP) is a genetically heterogeneous disease caused by mutations in many genes, including those encoding spastin, strumpellin, or REEP1. Allison et al. show that similar lysosomal phenotypes are associated with mutations in different classes of HSP proteins and suggest that defective ER–endosome contacts and endosome tubule fission may be a common cause of axon degeneration in the disease.

## Introduction

The ER consists of a series of sheets and dynamic tubules. The tubules make functionally important contacts with other organelles, including endosomes, mitochondria, and the plasma membrane (Raiborg et al., 2015). Contacts with endosomes are extensive, dynamic, and typically associated with microtubules (Friedman et al., 2013). They have been implicated in important cellular functions, including in fission of tubules from the endosomal body (Rowland et al., 2014). Endosomal tubules originate from early and late endosomes and sort receptors, such as the transferrin (TfnR) and mannose 6-phosphate (M6PR) receptors, for recycling away from the degradative lysosomal pathway (Maxfield and McGraw, 2004). The molecular machinery underlying the establishment and breakage of these fission-related ER–endosome contact sites is not completely understood, although the ER protein VAP has been implicated, via a mechanism that involves regulating endosomal phosphatidylinositol 4-phosphate levels and thereby the function of the WASH complex, an actin nucleating machinery that promotes endosomal tubule fission (Dong et al., 2016).

Previously, we proposed that efficient endosomal tubule fission requires the microtubule-severing ATPase spastin, as cells lacking spastin had increased endosomal tubulation coupled with defective TfnR recycling (Allison et al., 2013). However, it is not known whether spastin promotes ER-associated endosomal tubule fission or a distinct fission reaction not involving the ER. Rescue of the endosomal tubulation phenotype required spastin’s microtubule-severing ATPase capacity and its ability to bind the endosomal proteins IST1 and CHMP1B, components of the endosomal sorting complex required for transport (ESCRT)-III machinery (Allison et al., 2013). Because we also observed increased endosomal tubulation in cells lacking IST1, we suggested that IST1 is a key endosomal protein coordinating spastin’s role in tubule fission (Allison et al., 2013). Consistent with this, IST1 and CHMP1B have been proposed to form a helical complex involved in scission of tubular membranes (McCullough et al., 2015).

Autosomal dominant mutations in the gene encoding spastin (SPAST/SPG4) cause hereditary spastic paraplegia (HSP), a disease characterized by axonal degeneration in the central motor tracts. They are the single most common cause of the disease, being found in ∼40% of autosomal dominant HSP families (Blackstone et al., 2011). Study of HSPs has informed the molecular pathology of axonopathy, a process contributing to common neurological disorders, including Alzheimer dementia and multiple sclerosis. Of >70 known genes mutated in HSP (Hensiek et al., 2015), most encode proteins functioning in membrane traffic/modeling, with subsets of these involved in ER shaping (including those associated with the most common forms of HSP: spastin, atlastin-1, and REEP1), endosomal tubule fission (including the WASH complex member strumpellin as well as spastin), and lysosomal biogenesis and function (including SPG11, SPG15, and AP5 complex members) (Harbour et al., 2010; Park et al., 2010; Blackstone et al., 2011; Montenegro et al., 2012; Allison et al., 2013; Chang et al., 2014; Renvoisé et al., 2014; Hirst et al., 2015; Raza et al., 2015; Varga et al., 2015). No mechanism linking these subsets into a unifying disease pathway is known, although spastin has been implicated in two of these processes, hinting that there may be some connection.

Here we investigate the role of spastin in endosomal tubule fission and examine consequences of failure of this process. We show that endosomal IST1 and an ER-localized isoform of spastin (M1-spastin) interact at ER–endosome contacts to promote endosomal tubule fission. When this fails because of lack or abnormality of spastin, defects in M6PR sorting cause defective lysosomal enzyme traffic accompanied by abnormal lysosomal morphology, including in primary cortical neurons from a spastin-HSP mouse model, patient fibroblasts, and induced pluripotent stem cell (iPSC)-derived patient neurons. Consistent with a critical role for ER-mediated endosomal tubule fission in controlling lysosome function, we observed strikingly similar lysosomal abnormalities in cells lacking another HSP protein, the WASH component strumpellin, and in mouse neurons lacking the ER-shaping HSP protein REEP1. Thus as well as characterizing molecular machinery that drives ER-mediated endosomal tubule fission and linking a disease process to ER–endosome contact machinery, by coupling ER-mediated endosomal tubule fission to lysosome function, we have uncovered a unifying pathway that links several groups of HSP proteins previously considered distinct and includes the most common subtypes of HSP. We propose that this is a central pathway of HSP pathogenesis.

## Results

### Spastin promotes endosomal tubule fission

**Figure 1.**
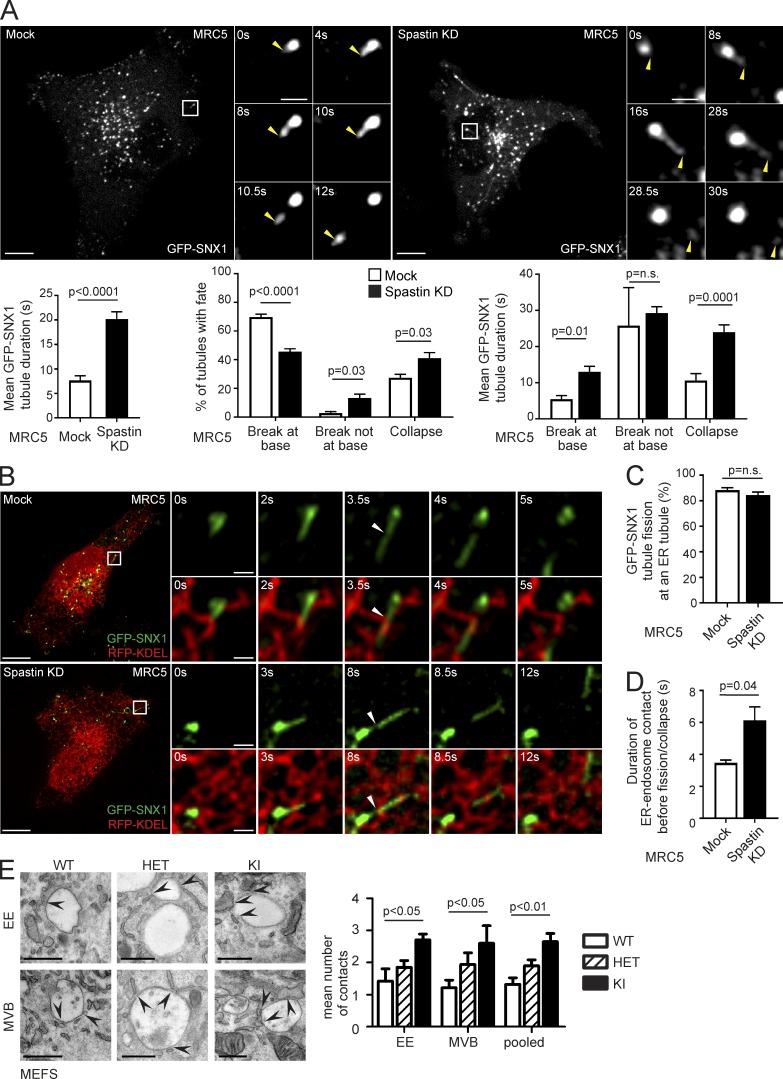
**Spastin promotes endosomal tubule fission at ER tubules.** (A) Stills from Video 1 of MRC5 cells stably expressing GFP-SNX1, showing tubule fission in control cell or cell depleted of spastin by siRNA knockdown (KD). The main panels show an overview of the cell; the small panels illustrate a single fission event. Yellow arrowheads track the tip of the elongating endosomal tubule and the fissioned domain. Time 0 corresponds to the frame in which the tubules first emerge. Corresponding histograms show mean tubule duration (left), mean percentage of tubules with different fates (middle), and duration of tubules with each fate (right). left, *n* = 6 experiments; middle and right, *n* = 5; see Materials and methods for numbers of tubules analyzed in these and subsequent live cell experiments. Bars in small panels, 2 µm. (B) Stills from Video 3 of MRC5 cells stably expressing GFP-SNX1 and transiently expressing RFP-KDEL, showing mock-transfected (top) or spastin KD (bottom) cell. The main panels show an overview of the cell; the small panels illustrate a single fission event. White arrowhead marks approximate site of fission. Time 0 corresponds to the frame in which the tubules first emerge. Bars in small panels, 1 μm. The corresponding histograms show percentage of fission events occurring at site of ER tubule overlap (C) and the duration of overlap between ER and endosomal tubule before breakage or collapse (D); *n* = 3 experiments. (E) EM of early endosome (EE) and multivesicular body (MVB) structures in MEFs from spastin^wt/wt^ (WT), spastin^wt/N384K^ (HET), and spastin^N384K/N384K^ (KI) animals. Arrows indicate points of ER contact, and the mean number of contacts is quantified (pooled, EE and MVB results combined). *n* = 3 experimental repeats. See Materials and methods for number of structures analyzed. Bars: (light micrographs, large panels) 10 µm; (EM) 500 nm. All histograms show mean ± SEM. P-values generated by two-tailed Student’s *t* test (A, C, and D) or ANOVA for effect of genotype (E).

Our previous observations suggested that spastin promotes endosomal tubule fission, but we had not proven this directly (Allison et al., 2013). To address this, we performed live-cell microscopy in human fibroblasts (MRC5 cells, whose flat morphology is amenable to live-cell imaging) stably expressing the endosomal tubular marker GFP-SNX1 (Fig. 1 A, Fig. S1 A, and Video 1). In cells depleted of spastin by siRNA transfection, mean GFP-SNX1 tubule duration was increased (Fig. 1 A, bottom left; and Fig. S1 B). We classified the fates of individual tubules into three categories; fission near the tubule base (within 1.5 µm of endosomal body), fission elsewhere on the tubule, and collapse into the endosomal body without breakage (likely representing failure of fission). In cells lacking spastin, the proportion of tubules that broke at the base was reduced, whereas the proportion that collapsed or broke elsewhere on the tubule was increased (Fig. 1 A, bottom middle). The duration of tubules undergoing these distinct fates was longer in cells lacking spastin (Fig. 1 A, bottom right). Consistent with these findings, in fixed MRC5 cells lacking spastin, a significantly increased percentage of cells had endogenous SNX1 tubules >2 µm long (Fig. S1 C).

We examined the physiological relevance of these findings using murine embryonic fibroblasts (MEFs) from a knock-in mouse model expressing, at physiological levels, spastin incorporating the N384K mutation (equivalent to human disease mutation N386K), rendering the protein ATPase defective and so unable to sever microtubules. The mice develop a gait and axonal phenotype consistent with HSP (Connell et al., 2016). In fixed spastin^N384K/N384K^ MEFs, we saw an increased percentage of cells with endogenous SNX1 tubules >2 µm long (Fig. S1 D). Live-cell experiments using GFP-SNX1 stably expressed at subendogenous levels demonstrated increased tubule duration in spastin^N384K/N384K^ MEFs, with fewer tubules breaking near the endosomal body, more breaking at other points along the tubule, and a trend toward increased tubule collapse (Video 2; and Fig. S1, A and E). We concluded that spastin is required for efficient endosomal tubule fission, and this function requires its ATPase activity.

### Spastin regulates ER-associated endosomal tubule fission

Endosomal tubule fission has been reported to occur at sites of ER contact in ∼80% of cases (Rowland et al., 2014). However, the specific relationship between SNX1 tubule fission and the ER, and the role of spastin in this process, has not been determined. We imaged living MRC5 cells stably expressing GFP-SNX1 at near-endogenous levels (Fig. S1 A) and transiently expressing the ER marker RFP-KDEL. In both wild-type and spastin-depleted cells, >80% of endosomal tubules that arose in an area of resolvable ER broke at a point where they crossed an ER tubule (Fig. 1, B and C; and Video 3). The duration of apparent contact between ER and endosomal tubules from establishment until fission or collapse was longer in cells lacking spastin (Fig. 1 D). EM demonstrated a significant increase in the number of ER contacts (apposing membranes within 20 nm) with early and late endosomes in spastin^N384K/N384K^ MEFs and HeLa cells depleted of spastin (Figs. 1 E and S1 F), consistent with the idea that ER–endosome contacts are broken by spastin-mediated endosomal tubule fission. Considered together, our observations indicate that spastin drives ER-associated endosomal tubule fission.

### IST1–spastin interaction links endosomes to the ER

Spastin has two main isoforms, M1-spastin and M87-spastin (Fig. S2 A; Claudiani et al., 2005). M1-spastin localizes to the ER at steady state, where it interacts, via a hydrophobic region not present in M87-spastin, with ER-shaping proteins of the REEP, reticulon, and atlastin families, including several implicated in HSP (Sanderson et al., 2006; Connell et al., 2009; Park et al., 2010; Montenegro et al., 2012). In contrast, M87-spastin is predominantly cytosolic but dynamically recruited to endosomes by interactions with the ESCRT-III proteins, notably CHMP1B and IST1 (Reid et al., 2005; Agromayor et al., 2009; Connell et al., 2009; Renvoisé et al., 2010). We showed previously that M87-spastin rescues the endosomal tubule fission defect in cells lacking endogenous spastin. Surprisingly, in view of its ER localization, small amounts of M1-spastin were also sufficient to rescue the endosomal tubule fission defect (Allison et al., 2013). The discovery of ER-associated endosomal tubule fission provides a possible explanation for this: M1-spastin functions at ER contact sites involved in endosomal tubule fission.

Because we had already determined that IST1 is a key endosomal protein coordinating spastin’s role in endosomal tubulation in HeLa cells (Allison et al., 2013), we began by examining whether IST1–M1-spastin interaction defined a set of ER–endosome contact sites involved in endosomal tubule fission. We first formally verified that the increased endosomal tubulation observed in cells lacking IST1 represented defective tubule fission, quantifying the fate and duration of GFP-SNX1 endosomal tubules in MRC5 cells. We found results similar to those for spastin, with an increased tubule duration and decreased breakage near the tubule base in cells lacking IST1 (Video 4 and Fig. S2 B).

**Figure 2.**
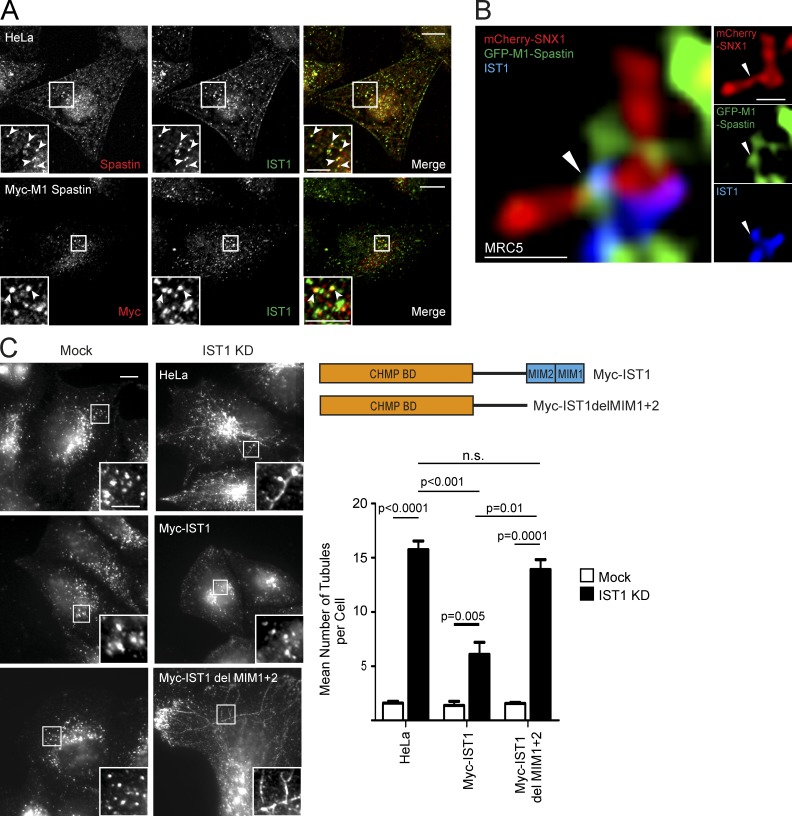
**Spastin–IST1 interaction drives endosomal tubule fission.** (A) Wild-type HeLa cells or HeLa cells stably expressing Myc-M1-spastin were imaged by confocal immunofluorescence microscopy (IF) with the antibodies indicated. Arrowheads indicate colocalized puncta. In cells labeled with endogenous spastin, soluble cytosolic signal was removed using a prefixation cytosol extraction buffer. (B) MRC5 cells stably expressing GFP-M1 spastin and mCherry-SNX1 were labeled with IST1 and GFP antibodies and imaged with Airyscan IF. Small panels show individual channels. Arrowheads indicate a region of colocalization between IST1 and spastin at the base of a SNX1 tubule. (C) Wild-type HeLa cells or cells expressing the siRNA-resistant proteins indicated (see schematic) were subjected to endogenous IST1 KD, then fixed and visualized by IF for endogenous SNX1. Mean ± SEM number per cell of SNX1 tubules >2 µm long is shown in the histogram (*n* = 5). Bars: (A and C, main image) 10 µm; (A and C, magnified insets) 5 µm; (B) 500 nm. P-values generated by two-tailed Student’s *t* test.

IST1 has been reported to localize to an endosomal subdomain juxtaposed to SNX1 (Agromayor et al., 2009), and consistent with this, we saw colocalization of IST1 with endosomal markers and close proximity to SNX1 signal in HeLa or MRC5 cells (Fig. S2 C). Furthermore, in HeLa cells, we observed punctate colocalization between endogenous spastin and IST1 and between stably expressed Myc-M1-spastin and IST1 (Fig. 2 A). Consistent with the idea that spastin and IST1 function to promote endosomal tubule fission, using superresolution Airyscan microscopy in MRC5 cells, we detected colocalization between stably expressed GFP-M1-spastin and endogenous IST1 on endosomal tubules labeled by mCherry-SNX1 (Fig. 2 B). This colocalization was often, but not always, located close to the base of the SNX1 tubule and was observed at points of tubule constriction (Fig. 2 B, Fig. S2 D, and Video 5). In the context of the well-validated IST1–spastin interaction, we concluded that the ER-localized M1-spastin isoform interacts with endosomal IST1 on SNX1 tubules (Agromayor et al., 2009; Renvoisé et al., 2010).

We next examined what happens to endosomal tubule fission when IST1–spastin binding is disrupted. Spastin’s MIT domain interacts with MIT-interacting motifs (MIMs) in the C terminus of IST1 (Agromayor et al., 2009; Renvoisé et al., 2010). Consistent with our previous demonstration that spastin’s regulation of endosomal tubule fission requires an MIT domain capable of interacting with ESCRT-III (Allison et al., 2013), siRNA rescue experiments showed that only IST1 containing the MIMs could rescue the increased endosomal tubulation seen in cells lacking endogenous IST1 (Figs. 2 C and S2 E). Considered together, our experiments indicate that IST1 and M1-spastin interact at ER–endosome contact sites, and that this interaction is required for efficient ER-mediated fission of endosomal tubules.

### Spastin and IST1 promote endosome-to-Golgi traffic

**Figure 3.**
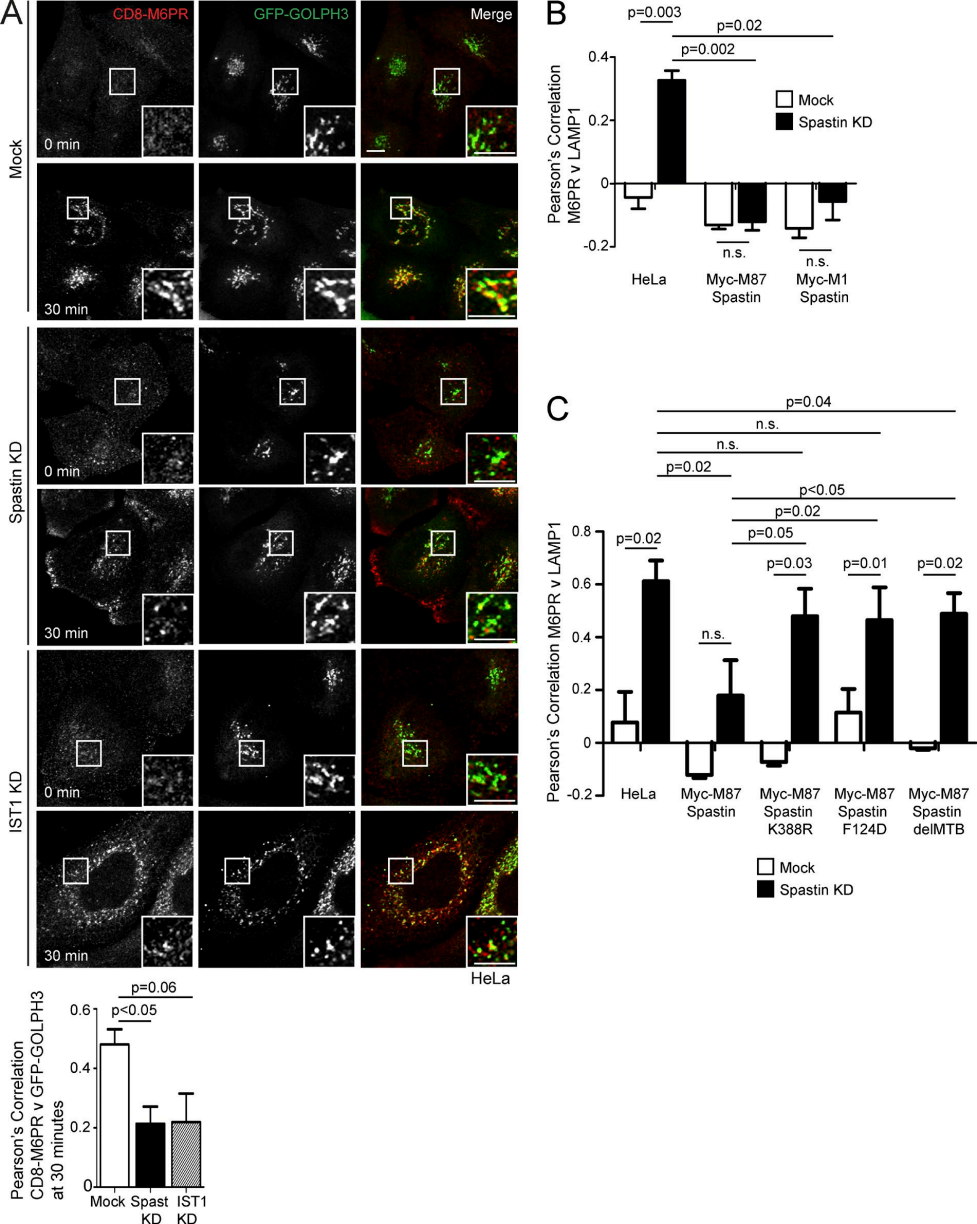
**Spastin and IST1 regulate endosome-to-Golgi traffic.** (A) Anti-CD8 antibody uptake experiments in HeLa cells stably expressing CD8-ciM6PR and GFP-GOLPH3 (a Golgi marker), and subjected to IST1 or spastin KD. Cells were fixed at the times indicated, and CD8 and GFP signal were visualized by confocal immunofluorescence microscopy. Mean ± SEM colocalization between the markers (Pearson’s correlation) at 30 min is shown in the histogram (*n* = 6). Bars, 10 µm. (B and C) Wild-type HeLa cells or HeLa cells stably expressing the siRNA-resistant spastin proteins indicated were subjected to endogenous spastin KD, fixed, and visualized for M6PR and LAMP1. Mean ± SEM colocalization was plotted. *n* = 4 (B) or 5 (C). In B and C, p-values between the HeLa mock and all other mock conditions were not significant. P-values generated by two-tailed Student’s *t* test.

M6PRs capture M6P-tagged lysosomal enzymes at the TGN and traffic them to endosomes. After cargo release, M6PRs are retrieved back to the TGN, via interaction with the retromer complex, in SNX1/SNX2-positive endosomal tubules (Carlton et al., 2004; Seaman, 2012). To understand whether spastin-driven endosomal tubule fission is required for this retrieval, we used a HeLa line stably expressing a reporter protein comprising CD8 fused to the cytoplasmic tail of the cation-independent M6PR (ciM6PR; Seaman, 2007). As some CD8-M6PR fusion protein is present at the plasma membrane and subject to endocytosis and subsequent trafficking, anti-CD8 antibody uptake experiments reveal the dynamics of endosome to TGN M6PR traffic. In wild-type cells, we found strong colocalization between the Golgi marker GOLPH3 and CD8-M6PR 30 min after uptake (Fig. 3 A). In cells lacking spastin, colocalization between GOLPH3 and CD8-M6PR was reduced (Fig. 3 A), indicating that spastin is required for efficient endosome-to-Golgi traffic of M6PRs. Cells lacking IST1 showed a similar trend (Fig. 3 A).

Failure to sort M6PRs away from endosomes may result in increased colocalization of M6PR with lysosomal markers. In HeLa cells lacking spastin, colocalization between ciM6PR and the lysosomal marker LAMP1 was significantly increased (Figs. 3 B and S3 A). This phenotype was rescued by both wild-type M1-spastin and M87-spastin (Fig. 3 B and Fig. S3, A and B). However, forms of spastin engineered to block interaction with ESCRT-III (by F124D mutation) or microtubules (by deletion of the MTB), or made unable to hydrolyze ATP and sever microtubules (by K388R mutation), gave minimal or no rescue (Fig. 3 C and Fig. S3, C and D; Evans et al., 2005; White et al., 2007; Yang et al., 2008). These results are consistent with the properties of spastin that promote endosomal tubule fission (Allison et al., 2013).

### Spastin controls lysosomal morphology

**Figure 4.**
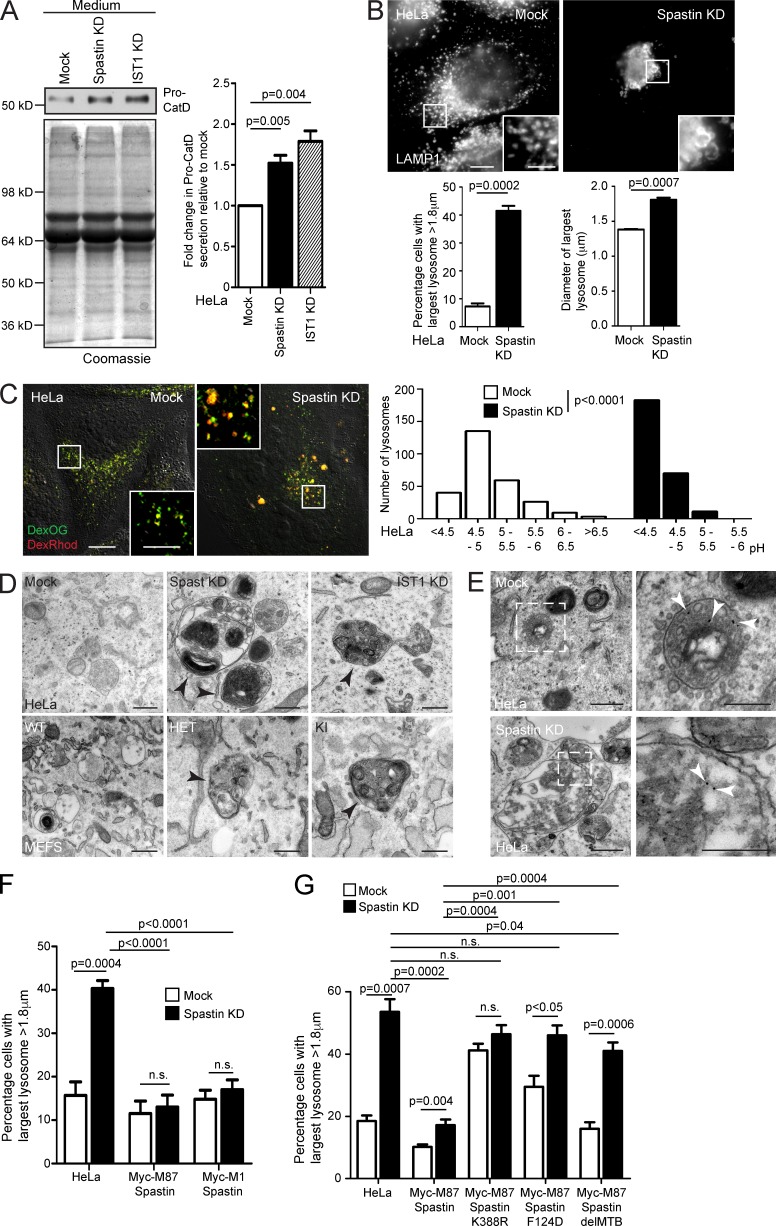
**Spastin and IST1 regulate lysosomal enzyme traffic and lysosome morphology.** (A) HeLa cells were subjected to spastin or IST1 KD, then protein precipitated from collected culture medium was immunoblotted for pro–cathepsin D (Pro-CatD). Coomassie staining validates equal loading. Blots were quantified, and the mean ± SEM fold-change in Pro-CatD secretion was plotted (*n* = 5). (B) HeLa cells were subjected to spastin KD, fixed, and labeled for LAMP1, then the diameter of the largest lysosome was measured in 100 cells/condition. The mean ± SEM percentage of cells with largest lysosome >1.8 µm and mean diameter of the largest lysosome are shown (*n* = 4). (C) HeLa cells were subjected to spastin KD and incubated in culture medium with dextran-Oregon Green (DexOG; fluorescence is acid quenched) or dextran-tetramethylrhodamine (DexRhod; fluorescence is pH insensitive) for a 4-h pulse, which was chased into the terminal degradative compartment for 20 h with dextran-free medium. Living cells were then imaged, and the ratio of red to green pixels in 300 labeled puncta was measured and used to calculate pH, as described in Materials and methods. In the image, the differential interference contrast channel is shown to allow definition of the cell boundaries. The pH distribution of puncta from a single experiment is shown in the corresponding histograms. (D) Top, EM of HeLa cells subjected to spastin or IST1 KD, showing abnormal endolysosomal structures with abnormal dense membrane (arrowheads). Bottom, EM of MEFs from spastin^N384K^ mice with genotypes indicated. Abnormal lysosomes indicated by arrowheads. (E) EM after BSA-gold uptake in mock-transfected or spastin-depleted HeLa cells. Right panels show the boxed areas indicated in the left panels; gold indicated by arrowheads. (F and G) Wild-type HeLa cells or HeLa cells stably expressing the siRNA-resistant spastin proteins indicated were subjected to endogenous spastin KD, fixed, and labeled for LAMP1. Mean ± SEM percentage of cells with largest lysosome >1.8 µm is plotted (*n* = 6, 100 cells/condition in each). All p-values generated by two-tailed Student’s *t* test, except C, in which differences in pH were analyzed using a two-tailed Mann–Whitney *U* test. Bars in light micrographs: (main image) 10 µm; (magnified insets) 5 µm. Bars in EM: 500 nm; (magnified images in E) 250 nm.

Defective endosome-to-Golgi M6PR traffic typically reduces availability of M6PRs at the TGN, resulting in reduced capture and traffic to endosomes of M6P-tagged lysosomal enzymes, which are instead secreted. Consistent with this, secretion of the lysosomal enzyme cathepsin D was increased in cells lacking spastin or IST1 (Figs. 4 A and S4 A). We predicted that this abnormal enzyme sorting would cause a lysosomal defect. In HeLa cells lacking spastin or IST1, and in spastin^N384K/N384K^ MEFs, we saw an increase in the proportion of cells with large LAMP1-positive lysosomes (Figs. 4 B and S4 B).

We characterized the lysosomal phenotype in cells lacking spastin in more detail. We quantified the overall size distribution of lysosomes in HeLa cells, visualizing the terminal degradative compartment by imaging fluorescent puncta 20 h after a 4-h incubation with fluorescent dextran. The mean surface area of individual lysosomes was significantly greater in spastin-depleted cells (Fig. S4 C; mean ± SEM: mock, 0.78 ± 0.018 µm^2^; spastin-depleted, 0.88 ± 0.023 µm^2^). However, this difference was predominantly caused by an increased number of very large lysosomes in spastin-depleted cells; when lysosomes with a surface area >2.5 µm^2^ (equivalent to a diameter of 1.8 µm, assuming a circular shape) were excluded, there was no significant difference between wild-type and spastin-depleted cells (mock, 0.73 ± 0.015 µm^2^; spastin-depleted, 0.76 ± 0.017 µm^2^). Cells lacking spastin also had a moderate but significant reduction in the number of lysosomes per cell (Fig. S4 D), but there was no significant difference in the percentage of lysosomes that were located in the center of the cell versus the periphery (defined as within 5 µm of the cell edge; Fig. S4 E). We determined whether spastin depletion affected lysosomal pH by a quantitative ratiometric fluorescent microscopic method involving uptake into the terminal lysosomal compartment of separate pH-quenchable and pH-insensitive fluorescently labeled dextrans (Bright et al., 2016; Johnson et al., 2016). HeLa cells lacking spastin had a strikingly reduced lysosomal pH, with a mean pH of 4.3, versus pH 5.2 in wild-type cells (Fig. 4 C), underscoring functional differences caused by spastin depletion.

We examined lysosomal ultrastructure. In HeLa cells lacking spastin or IST1, and in spastin^N384K/N384K^ MEFs, we found highly abnormal endolysosomes and lysosomes that had a spectrum of abnormal appearances, including enlarged organelles with internal membrane that was tightly or more loosely packed into linear or circular stacks or coils, organelles with a dense network or honeycomb appearance, and more vacuolar structures, which contained only a few intraluminal vesicles or membranes. Organelles with intermediate appearances were observed, suggesting a continuous spectrum of abnormality. Approximately 50% of lysosomes had abnormal morphology on this spectrum (Figs. 4 D and S4 F). These abnormal lysosomes could be very large, but smaller organelles also frequently had abnormal morphology (Fig. S4 F). BSA-gold uptake confirmed that these abnormal structures had endosomal origin (Fig. 4 E).

We performed siRNA rescue experiments in HeLa cells to determine the properties of spastin required to regulate lysosomal size. M1-spastin or M87-spastin was sufficient to rescue lysosomal enlargement, and the rescue required the ability to hydrolyze ATP and bind ESCRT-III and microtubules (Fig. 4, F and G; and Fig. S5, A and B). Rescue experiments with IST1 indicated that the spastin-binding MIMs were important for rescue of lysosomal size (Fig. S5 C). We concluded that similar functional properties, including the ability to interact with IST1 and sever microtubules, are necessary for spastin to regulate both lysosomal morphology and endosomal tubule fission, consistent with the idea that defective endosomal tubule fission causes the lysosomal abnormality.

### Lysosomal defects in spastin-HSP neuronal models

**Figure 5.**
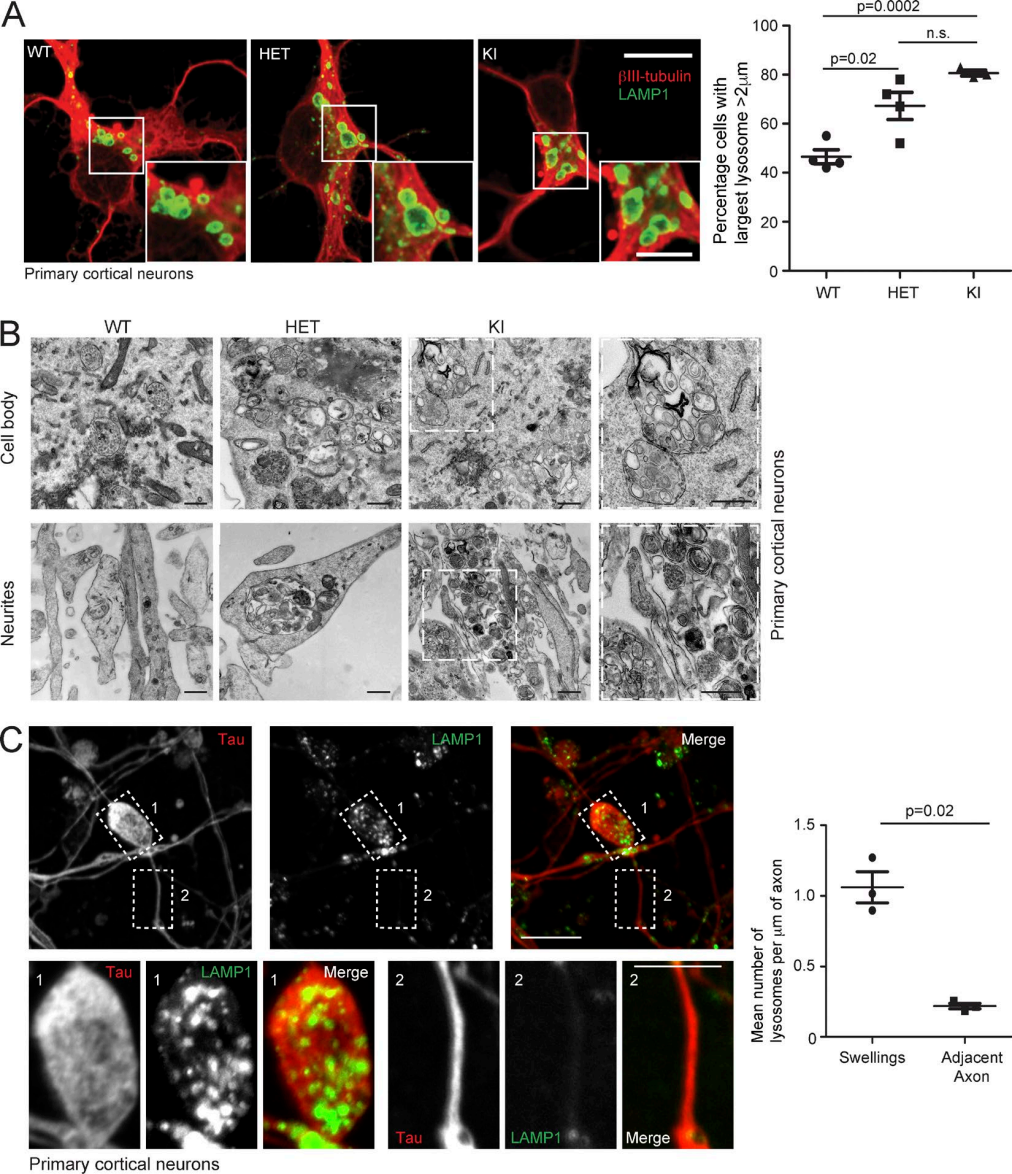
**Abnormal lysosomes in spastin-HSP mouse neurons.** (A) Primary cortical neurons derived from spastin^wt/wt^ (WT), spastin^wt/N384K^ (HET), and spastin^N384K/N384K^ (KI) animals were processed for immunofluorescence microscopy with the neuronal marker βIII-tubulin (red) and LAMP1 (green). The percentage of cells with largest lysosome >2 µm diameter was plotted for each animal (100 cells/animal). Bars indicate mean ± SEM. (B) EM of primary neuron cell bodies and neurites from animals with spastin^N384K^ genotypes indicated. Boxed areas are in higher magnification on the right. (C) Confocal immunofluorescence microscopy (IF) of axonal swelling in spastin^N384K/N384K^ mouse primary cortical neuron, labeled for LAMP1 and the axonal marker tau. Boxed areas shown in higher power in bottom panels. The mean ± SEM number of LAMP1-positive vesicles/µm of axonal length in 20 swellings versus adjacent axonal segments is plotted for three animals. Bars: (EM) 500 nm; (IF main panels) 10 µm; (IF magnified insets) 5 µm. P-values generated by two-tailed Student’s *t* test.

To explore the pathological relevance of our findings, we examined fixed primary cortical neurons, the cells that undergo axonopathy in HSP, and found an increased percentage of spastin^N384K/N384K^ neurons with large lysosomes versus wild-type cells (Fig. 5 A). In the cell body and neurites, lysosomes had abnormal ultrastructure, with many endolysosomal membrane coils and frequent gross enlargement (Fig. 5 B). Axons from spastin^N384K/N384K^ mice develop swellings, and these were significantly enriched for LAMP1-positive vesicles, compared with adjacent axonal segments of equivalent length (Fig. 5 C; Connell et al., 2016). Consistent with this, EM demonstrated that abnormal lysosomes often clustered in neurites (Fig. 5 B).

**Figure 6.**
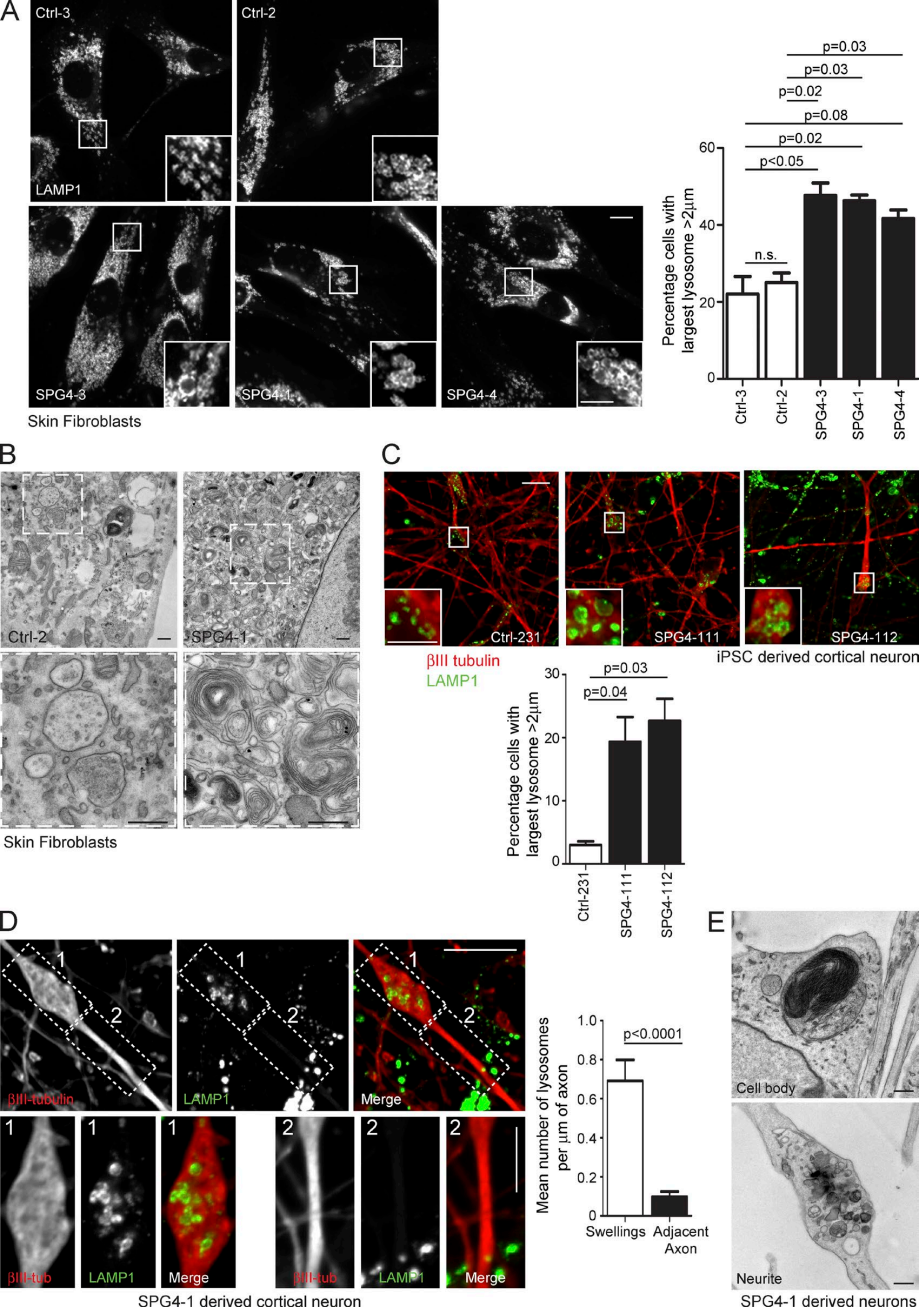
**Lysosomal abnormalities in human spastin-HSP patients.** (A) Skin fibroblasts from control subjects (top) or three spastin-HSP patients (SPG4; bottom) were fixed and visualized by confocal immunofluorescence microscopy (IF) for LAMP1. In each image, the inset panels show higher-magnification views of the boxed region. The diameter of the largest lysosome was measured in 100 fibroblast cells from each subject, and the mean percentage of cells with largest lysosome >2 µm was plotted in the corresponding histogram (*n* = 3 experimental repeats). Histogram shows mean ± SEM. (B) EM of fibroblasts from spastin-HSP patient and control (bottom, magnified views of boxed areas). (C) Cells from two human cortical neuron lines (SPG4-111 and 112) and a control line (Ctrl-231) were fixed and labeled for βIII-tubulin (as a marker of neuronal differentiation) and LAMP1. The diameter of the largest lysosome was measured in βIII-tubulin–positive cells, and the mean ± SEM percentage of neurons with largest lysosome >2 µm was plotted in the corresponding histogram (*n* = 3/line, 100 cells/repeat). The two SPG4 neuronal lines were differentiated from separate NPC cultures derived from patient SPG4-1 iPSCs. (D) Cells from two human cortical neuron lines (SPG4-111 and 112) were labeled for βIII-tubulin and LAMP1. The images show a typical axonal swelling. The number of lysosomes in swellings (box 1) and adjacent axonal segments (box 2) was counted and normalized for segment length, and the mean ± SEM number of LAMP1-positive vesicles/µm of axonal length are presented in the corresponding histogram (*n* = 20 swellings). (E) EM of lysosomal structures in patient SPG4-1 iPSC-derived cortical neurons. Bars: (EM) 500 nm; (IF main panels) 10 µm; (IF magnified insets) 5 µm. P-values generated by two-tailed Student’s *t* test.

To test whether lysosomal abnormality was a feature in human patients, we examined fibroblast lines derived from three SPG4 (spastin)-HSP subjects (see Materials and methods for patient details) and found a significant increase in the proportion of cells with large lysosomes (Fig. 6 A). These lysosomes had ultrastructural abnormalities highly reminiscent of those seen in the cell models described earlier (Fig. 6 B). We then differentiated human iPSCs reprogrammed from fibroblasts of SPG4-1 and Ctrl-2 into cortical neurons via neuronal precursor cell (NPC) cultures. We observed an increased percentage of neurons with enlarged lysosomes in cultures differentiated from two separate SPG4-1 NPC lines (Fig. 6 C). The axons of SPG4 patient-derived neurons develop swellings (Denton et al., 2014; Havlicek et al., 2014), and these were enriched for lysosomes (Fig. 6 D). Ultrastructural studies on neurons differentiated from another iPSC line from the same patient demonstrated enlarged lysosomes containing abnormal membrane accumulations (Fig. 6 E). Consistent with light microscopy findings, we observed clustered lysosomes containing accumulations of membrane in swollen sections of neurites (Fig. 6 E).

### Lysosomal abnormalities in other HSP subtypes

**Figure 7.**
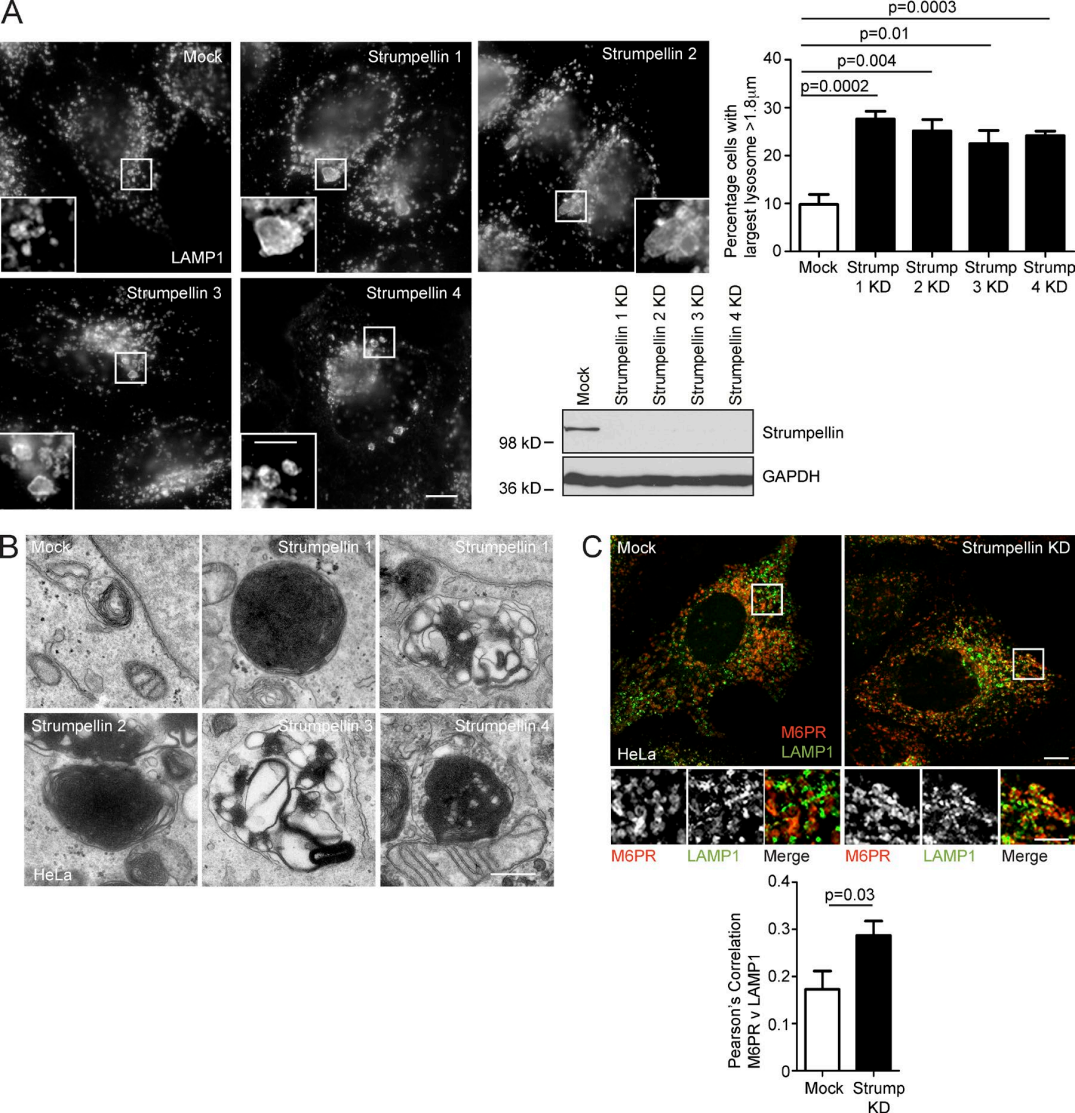
**Abnormal lysosomes in strumpellin HSP models.** (A) HeLa cells were depleted of strumpellin using four individual siRNAs (strumpellin 1–4), fixed, and labeled for LAMP1, then the diameter of the largest lysosome in each cell was measured. The mean ± SEM percentage of cells with a largest lysosome >1.8 µm is shown (*n* = 6 repeats, 100 cells/repeat). The immunoblot verifies strumpellin depletion. (B) EM of endolysosomal structures in wild-type HeLa cells (mock) or HeLa cells subjected to strumpellin depletion using siRNAs 1–4. With each of the siRNAs, abnormal endolysosomal structures encompassing a range of appearances, from those containing very dense networks of membrane to looser coils of membrane, were observed (compare the two images shown for strumpellin siRNA 1). (C) HeLa cells were mock-transfected or depleted with strumpellin siRNA, then fixed and visualized for M6PR and LAMP1. Colocalization between the two markers was quantified and plotted in the corresponding histogram (*n* = 3 repeats, 15 cells per condition in each repeat). Bars: (EM) 500 nm; (confocal immunofluorescence microscopy [IF] main panels) 10 µm; (IF magnified insets) 5 µm. P-values generated by two-tailed Student’s *t* test.

Our results indicate that spastin indirectly controls lysosomal morphology by regulating ER-associated endosomal tubule fission. We examined whether other HSP proteins potentially implicated in this process also affected lysosomal morphology. Mutations in the SPG8/KIAA0196 gene encoding strumpellin cause autosomal-dominant HSP (Valdmanis et al., 2007). The likely pathological mechanism is haplo-insufficiency, as the mutational spectrum includes a multiexon deletion and as no dominant-negative effects of mutant strumpellin have been identified in mammalian and zebrafish studies (Valdmanis et al., 2007; Freeman et al., 2013; Ishiura et al., 2014). Strumpellin participates in the WASH complex, which interacts with retromer and is required for efficient endosomal tubule fission. Consistent with this, cells depleted of strumpellin are known to have increased endosomal tubulation, including of SNX1 tubules (Derivery et al., 2009; Gomez and Billadeau, 2009; Harbour et al., 2010). Furthermore, correct function of the WASH complex in nucleation of actin on endosomes is regulated by ER–endosome contacts involving the ER-localized VAP proteins (Dong et al., 2016). Against this background, we predicted that cells lacking strumpellin would develop a lysosomal phenotype. Indeed, we found that an increased proportion of HeLa cells lacking strumpellin had enlarged lysosomes. We saw this phenotype when strumpellin was depleted with four separate siRNA oligonucleotides, indicating that it is highly unlikely to be caused by off-target siRNA effects (Fig. 7 A). The ultrastructure of endolysosomes in cells lacking strumpellin was strikingly similar to that observed in spastin-HSP models and ranged from organelles containing very tightly packed stacks or networks of membrane material to others in which the membranes were more loosely coiled (Fig. 7 B). Again, we saw these ultrastructural abnormalities in cells depleted of strumpellin using four separate siRNA oligonucleotides (Fig. 7 B). Finally, in view of the defective endosomal tubule fission and increased endosomal tubulation in cells lacking strumpellin, we predicted that M6PR sorting away from the endolysosomal compartment would be reduced by strumpellin depletion. Consistent with this, we found increased colocalization between M6PR and LAMP1 in cells depleted of strumpellin (Fig. 7 C). Thus lack of strumpellin, another HSP protein that promotes endosomal tubule fission, caused M6PR trafficking and lysosomal abnormalities highly reminiscent of those seen in spastin-HSP models.

**Figure 8.**
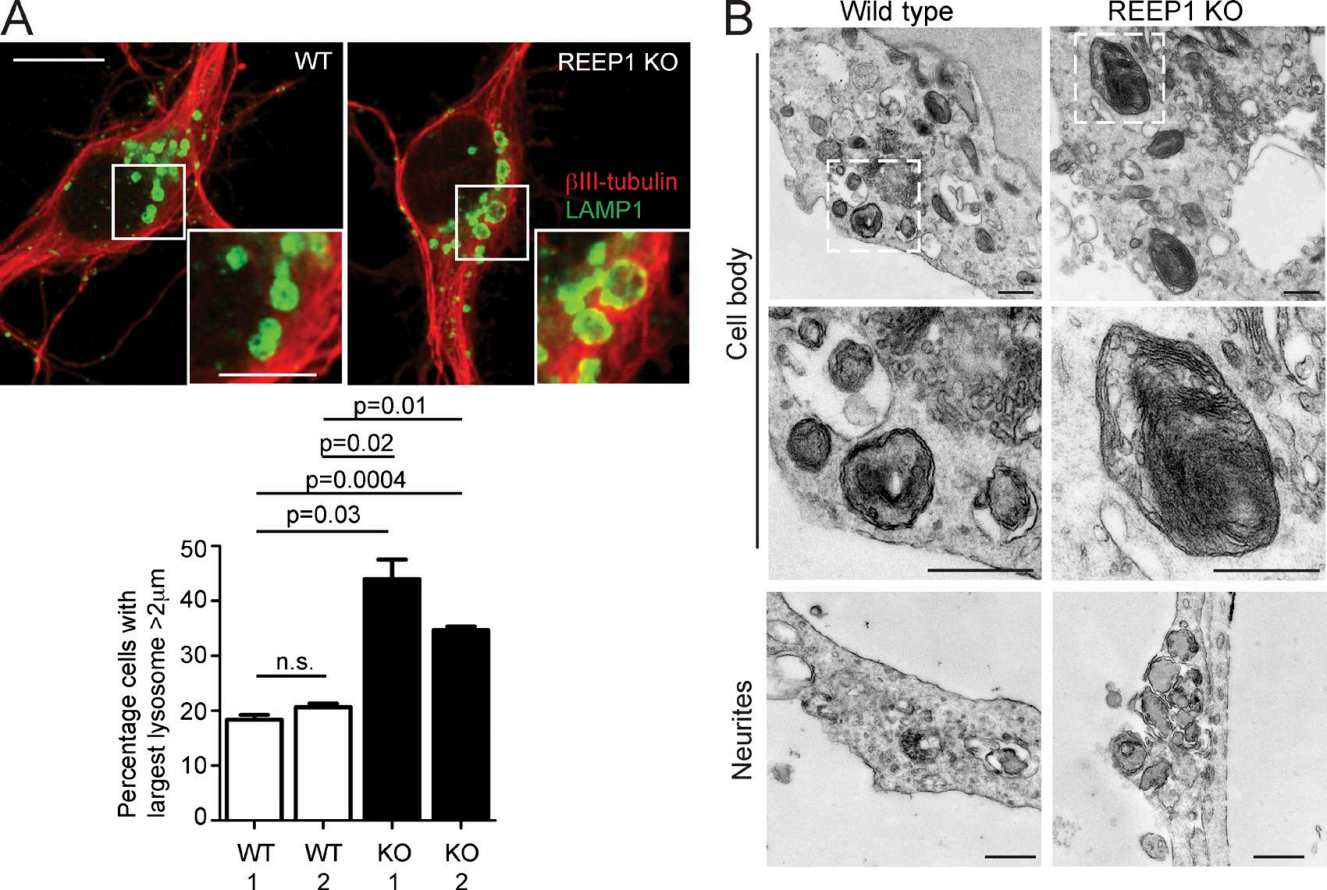
**Lysosomal abnormalities in REEP1 knockout mice.** (A) Primary cortical neurons from REEP1 KO or wild-type littermates were labeled with the neuronal marker βIII-tubulin (red) and LAMP1 (green). The diameter of the largest lysosome was measured, and the mean ± SEM percentage of cells with a largest lysosome >2 µm is shown (*n* = 3 repeats, at least 50 cells/repeat). (B) EM of primary cortical neurons from REEP1 KO mice or wild-type littermate controls. Middle panels are higher-magnification views of the boxed regions. Bars: (EM) 500 nm; (confocal immunofluorescence microscopy [IF] main panels) 10 µm; (IF magnified insets) 5 µm.

Alteration of ER morphology or dynamics by overexpression of an ER-shaping protein leads to defective ER-mediated endosomal fission (Rowland et al., 2014). We therefore predicted that abnormality of a classic ER-shaping HSP protein, REEP1, would cause a lysosomal phenotype. REEP1 interacts with M1-spastin, and loss-of-function mutations in the encoding gene cause autosomal dominant HSP (Beetz et al., 2008; Park et al., 2010; Hu et al., 2011). We examined cortical neurons from a REEP1-HSP knockout (KO) mouse model that develops behavioral and histological features compatible with HSP, and found an increase in the proportion of neurons with large lysosomes (Fig. 8 A; Beetz et al., 2013). The abnormal lysosomes had an ultrastructural appearance strikingly similar to those observed in spastin-HSP models, with the presence of stacked linear arrays of membranous material (Fig. 8 B). We concluded that lack of an ER-shaping HSP protein could also influence lysosomal morphology.

## Discussion

**Figure 9.**
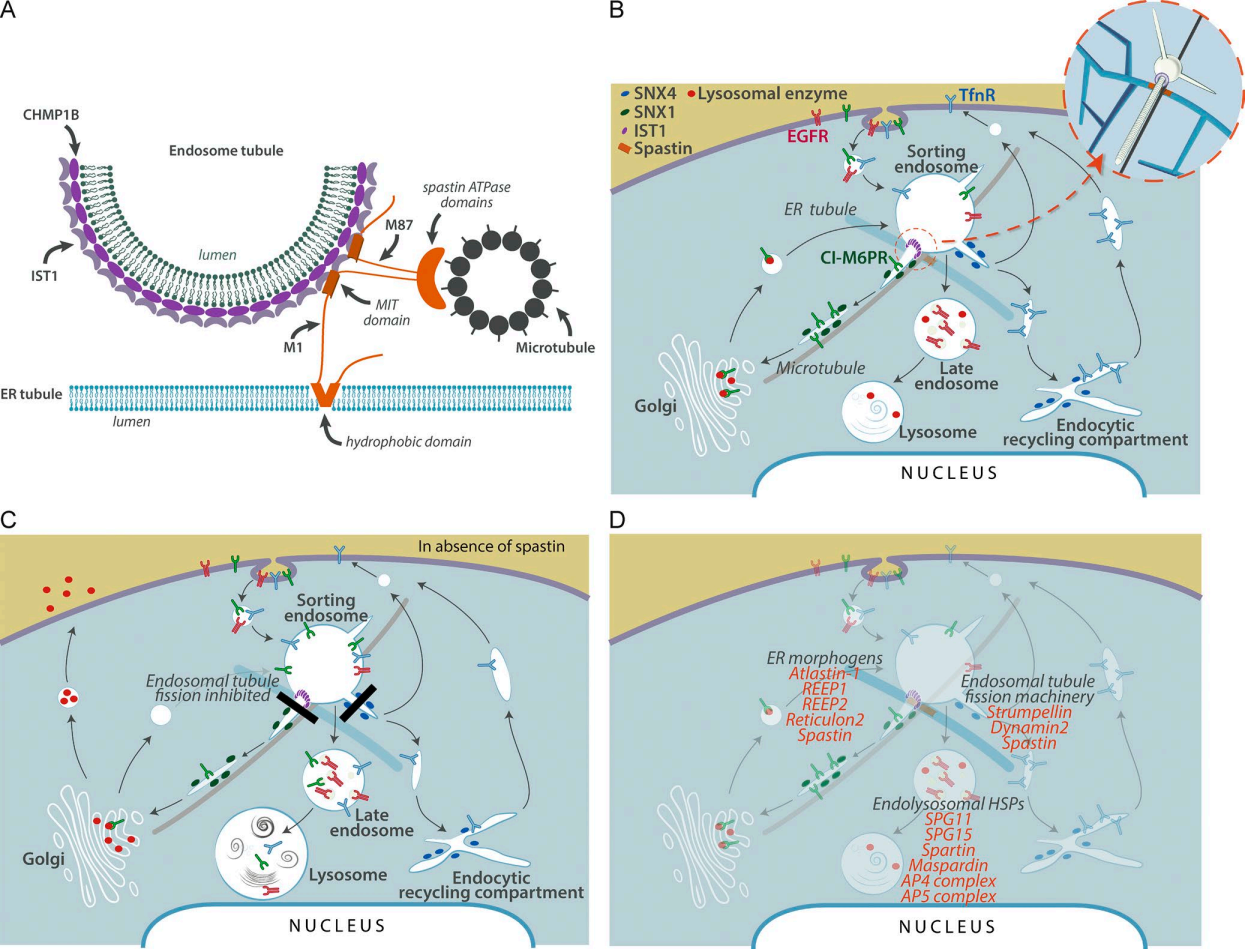
**Model for spastin mode of action and a proposed HSP cellular pathway.** (A) Schematic of the proposed ER–endosome contacts mediated by spastin and IST1. The CHMP1B and IST1 complex implicated in fission coats the endosomal tubule. A spastin hexameric complex is in orange, with the ATPase domains positioned to cut a microtubule, but for clarity the N-terminal tails of a single M87-spastin and M1-spastin molecule are shown. (B) Schematic diagram of key trafficking pathways influenced by spastin and IST1. Spastin and IST1 drive ER-mediated fission of tubules, decorated by sorting nexins, from the early sorting endosome, thus promoting endosome-to-Golgi traffic of ciM6PR and recycling of TfnR. CiM6PR then captures newly synthesized lysosomal enzymes at the Golgi, and the complex is then trafficked back to endosomes, to deliver lysosomal enzymes to the late endosomal/lysosomal degradative compartment. For simplicity, only a single ER tubule is shown. Inset, 3D view of the relationship between endosomal tubules, ER tubules, microtubules, and spastin (orange) and IST1 (purple). (C) In cells lacking spastin, fission of endosomal tubules is inhibited (black rectangles), so ciM6PR and TfnR are not efficiently sorted away from the endosome and instead are trafficked to the degradative compartment. Reduced availability of ciM6PR at the Golgi apparatus results in increased secretion and reduced delivery to the endosomal pathway of lysosomal enzymes. This causes lysosomal morphological and functional abnormalities, including increased lysosomal size and the presence of abnormal arrays of dense membrane. (D) Proposed sites of action of selected HSP proteins involved in membrane traffic, overlaid onto the trafficking pathways shown in B.

CHMP1B and IST1 have been proposed to form a helical complex on endosomal tubules, causing membrane constriction by promoting positive membrane curvature (McCullough et al., 2015). Our results suggest a model in which recruitment of spastin to this complex, via IST1–MIM-spastin MIT interaction, is critical for efficient breakage of endosomal tubules at ER-associated fission sites. Reconstructions of ER–endosome contacts demonstrate that they are frequently near microtubules (Friedman et al., 2013). As spastin’s ATPase activity, and hence ability to sever MTs, was required for endosomal tubule fission, our data best support the idea that severing of this subset of MTs is critical for the fission reaction (Fig. 9 A). We cannot fully exclude alternative models, e.g., where spastin drives fission by remodeling the CHMP1B-IST1 complex, analogous to VPS4’s role in remodeling ESCRT-III complexes (Henne et al., 2013).

This spastin-driven fission process is likely synergistic with the recently described function of VAP-A and -B, which act at ER–endosome contacts to regulate the WASH complex, thereby regulating fission of endosomal tubules (Dong et al., 2016). Indeed, the observation that both spastin and VAP proteins interact with protrudin, another protein present at ER–endosome contacts, suggests a mechanism by which these processes are coordinated (Mannan et al., 2006; Raiborg et al., 2015).

Lack of both M1- and M87-spastin is required to inhibit endosomal tubule fission, as either rescued increased endosomal tubulation in cells lacking endogenous spastin. M1-spastin is much less abundant than M87-spastin in all cells types. However, it is very efficient in promoting endosomal tubule fission: we obtained similar degrees of endosomal tubulation rescue with far lower concentrations of M1-spastin than M87-spastin (Allison et al., 2013). We suggest that this is because M1-spastin, being at the ER, is ideally placed to interact with IST1 at the base of the endosomal tubule where it contacts the ER. Spastin hexamerizes to sever microtubules, and because the two isoforms have the capacity to hetero-oligomerize, we propose that in the physiological situation, efficient hexamer formation involves nucleation by M1-spastin at the ER–endosome contact, followed by rapid recruitment of M87-spastin from the abundant cytosolic pool (Fig. 9 A; Roll-Mecak and Vale, 2008). Furthermore, the redundancy of M1- and M87-spastin in rescuing tubule fission provides an explanation for the observation that the vast majority of, if not all, spastin disease mutations affect both isoforms of the protein (Lumb et al., 2012).

Lysosomal abnormality, typically accompanied by lysosomal morphology remarkably similar to that observed in this study, has already been described in several rare HSP subtypes involving proteins that localize to endosomes, including SPG11, SPG15, and AP5 complex members (Khundadze et al., 2013; Renvoisé et al., 2014; Hirst et al., 2015; Varga et al., 2015). By showing that failure of ER-mediated endosomal tubule fission causes abnormal lysosomal morphology (Fig. 9, B and C), we now link HSP proteins that participate in this process to correct lysosomal function. As well as spastin and strumpellin, this potentially includes classic ER-shaping proteins, as correct ER morphogenesis also appears to be necessary for ER-mediated endosomal fission (Rowland et al., 2014). Consistent with this, we observed lysosomal abnormalities in cells lacking the archetypal ER-shaping protein REEP1. Because we found the lysosomal phenotype in cortical neurons from spastin and REEP1 mouse models, and in the case of spastin, from patient fibroblasts and iPSC-derived neurons, it is very likely relevant to the pathogenesis of HSP. Thus our work potentially links many classes of HSP protein previously considered functionally distinct, in a unifying pathway of HSP pathogenesis that incorporates all of the most common HSP subtypes, and which culminates in lysosomal dysfunction (Fig. 9 D). This is consistent with the idea that lysosomal dysfunction contributes to axonopathy in prevalent neurological diseases (Ashrafi et al., 2014; Gowrishankar et al., 2015; Xie et al., 2015) and suggests that insights from HSP will inform our knowledge of these common disorders. A key challenge now is to understand how lysosomal abnormality causes axonopathy. Lysosomal functions implicated in axonal disease, including autophagy or mitophagy, are strong pathological candidates (Ashrafi et al., 2014; Gowrishankar et al., 2015; Xie et al., 2015). Furthermore, lysosomal dysfunction may perturb lipid metabolism by inhibiting lysosomal degradation of membrane lipids or, by inhibiting receptor degradation, could disrupt key axonal signaling pathways such as bone morphogenetic protein signaling, both of which have already been implicated in HSP (Blackstone et al., 2011; Tesson et al., 2015).

## Materials and methods

### Antibodies

Rabbit polyclonal anti-spastin (86–340) (raised against a glutathione S-transferase fusion protein that incorporated residues 86–340 of M1-spastin) and anti-SNX1 (raised against a glutathione S-transferase protein fused to mouse SNX1) antibodies were produced as previously described (Seaman, 2004; Connell et al., 2009). Monoclonal anti-CD8 was produced by a mouse hybridoma cell line obtained from ATCC (CRL-8014), as described previously (Seaman, 2007). The SNX1 and CD8 antibodies were a gift of M. Seaman (University of Cambridge, Cambridge, England, UK). Mouse monoclonal anti-spastin (6C6) and α-tubulin (DM1A) and peroxidase-conjugated secondary antibodies for Western blotting were obtained from Sigma-Aldrich; mouse monoclonal anti-myc (4A6) and rabbit polyclonal anti–Cathepsin D (219361) from EMD Millipore; mouse monoclonal anti-TfnR (13-6800) from Invitrogen; rabbit polyclonal anti-IST1 (51002-1-AP) from Proteintech Group; mouse monoclonal anti-SNX1 (611482), anti-EEA1 (610456), and anti-Rab5 (610724) from BD transduction laboratories; rabbit polyclonal anti-GFP (ab6556), anti–βIII-tubulin (ab18207), mouse monoclonal anti-M6PR (ab2733), and anti–Tau-5 (ab80579) from Abcam; mouse monoclonal anti-LAMP1 (H4A3), rabbit polyclonal anti-strumpellin (C-14), and rat monoclonal anti-LAMP1 (1D4B) from Santa Cruz Biotechnology, Inc.; rabbit polyclonal anti-GAPDH (2118) from Cell Signaling Technology; and Alexa Fluor 488–, 568–, and 647–labeled secondary antibodies for immunofluorescence from Molecular Probes.

### Constructs

pLXIN-myc-Spastin and pLXIN-myc-IST1 constructs were generated as previously described (Allison et al., 2013). In brief, M87- or M1-spastin was cloned into the pIRESneo2 vector (NheI–BamHI; Takara Bio Inc.) followed by insertion of a myc tag (EcoRV–NheI), and then myc-spastin was further cloned into the pLXIN vector (SalI–BamHI; Takara Bio Inc.). Constructs were made resistant to spastin siRNA1 and 3 by introducing two mutations into each of the relevant sequences by site-directed mutagenesis. Functional mutant versions of pLXIN-myc-M87-spastin were then generated by site-directed mutagenesis. Codon-optimized IST1 isoform 1 (UniProt accession no. P53990) was synthesized by GenScript. A 5′ myc tag was added by insertion of IST1 into pIRESneo2-myc (NheI–BamHI). Codon optimization of the IST1 sequence rendered it resistant to both IST1 siRNA1. pBMN-GFP-M1-spastin was generated by cloning EGFP in-frame into pIRES-myc-M1-spastin and cloning GFP-M1-spastin from this vector into an empty pBMN vector. pLXIN-EGFP-SNX1 was generated by cloning EGFP-SNX1 from pIRES-EGFP-SNX1 (which encodes an N-terminally GFP-tagged murine SNX1 protein; a gift from M. Seaman) into the pLXIN vector (SalI–NotI). pLXIN-mCherry-SNX1 was generated by cloning mCherry into the GFP restriction sites used in the cloning of EGFP-SNX1 and pLXIN-EGFP-SNX1. RFP-KDEL (ss-RFP-KDEL) was generated as previously described (Altan-Bonnet et al., 2006). In brief, the first 30 aa of bovine preprolactin signal sequence were cloned into the Nhe1/Pst1 sites of pEGFP-C1 vector (removing the GFP sequence; Takara Bio Inc.). Monomeric RFP was mutagenized to insert a KDEL ER retention signal 5′ to the stop codon, then this was inserted into the prolactin signal sequence-containing vector (Sal1–BamH1). pEF321-T expressing SV40 large T antigen was a gift from S. Sugano (University of Tokyo, Japan).

### Stable cell lines

Stable cell lines were generated by retroviral transduction of HeLaM, MRC5, or mouse embryonic fibroblast (MEF) cells, using the spastin, IST1 and SNX1 pLXIN and pBMN constructs described earlier. A clonal HeLa cell line stably expressing GFP-GOLPH3 and CD8-ciM6PR (expressing CD8-tagged cytoplasmic tail of the bovine ciMPR and GFP-tagged human GOLPH3, at trans-Golgi network-resident protein) was a gift from M. Seaman (Seaman, 2004; Breusegem and Seaman, 2014). MEFs used to make GFP-SNX1 stable lines were immortalized by transfection with pEF321-T, expressing the SV40 large T antigen, and selecting for stable expression by cell survival over time.

### Cell culture

HeLaM and MRC5 cells were maintained as previously described (Connell et al., 2009). MEFs were prepared from embryonic day 14 (E14)–E15 mouse embryos and cultured in DMEM supplemented with penicillin/streptomycin (Sigma-Aldrich), l-glutamine (Sigma-Aldrich), minimal essential amino acids, and β-mercaptoethanol. Spastin mouse primary cortical neurons were obtained from E17 mouse embryos or day-old pups and cultured on poly-d-lysine–coated glass coverslips in neurobasal medium (Invitrogen) with B27 supplement (Invitrogen) and l-glutamine (Sigma-Aldrich). After 7 d in culture, neurons were fixed in 3.7% formaldehyde and processed for immunofluorescence microscopy. (Connell et al., 2016). REEP1 KO primary neurons were obtained from the cortices of P1 animals and grown on coverslips for 7 d. Cells were washed once in PBS and fixed in 4% (vol/vol) formaldehyde.

HeLaM, MRC5, and MEF cells stably expressing spastin, IST1, or EGFP-SNX1 constructs were additionally cultured in the presence of 500 µg/ml Geneticin (Invitrogen). HeLaM cells stably expressing GFP-GOLPH3 and CD8-ciM6PR were cultured in the presence of both 500 µg/ml Geneticin (Invitrogen) and 1 µg/ml puromycin (Sigma-Aldrich). MRC5 cells stably expressing GFP-M1-spastin and mCherry-SNX1 were cultured in the presence of both 500 µg/ml Geneticin (Invitrogen) and 200 µg/ml Hygromycin B (Thermo Fisher Scientific). Patient fibroblasts were cultured in IMDM/Glutamax containing 15% FBS (Invitrogen) and 1× penicillin/streptomycin (Invitrogen; Havlicek et al., 2014).

### Patients and fibroblast derivation

The patients included were whites with typical characteristics of pure spastic paraplegia and mutations in SPAST. The controls were unrelated healthy whites with no history of neurological disease. Human fibroblasts were obtained from dermal punch biopsies from the upper arm, after Institutional Review Board approval (4120) and written informed consent, at the movement disorder clinic at the Department of Molecular Neurology, Universitätsklinikum Erlangen (Erlangen, Germany).

Further details of the patients and cell lines derived from them are as follows: fibroblast line SPG4-3, derived from 48-y-old male patient with SPAST mutation c.139A>T, p.Lys47X; fibroblast line SPG4-4, derived from 50-y-old female patient with mutation involving deletion of exons 1–17 of SPAST; fibroblast line SPG4-1, derived from 51-y-old female patient with SPAST mutation c.1684C>T, p.Arg562X (this line was used to derive NPC/neuron lines SPG4-111, SPG4-112, and SPG4-123); ctrl-3, derived from a healthy 41-y-old male control patient; and ctrl-2, derived from a healthy 45-y-old female patient (this line was used to derive NPC/neuron line Ctrl-231).

### Animals

Mice were maintained in accordance with UK and European Union regulations. Animal work performed in the UK for this study was approved by the University of Cambridge Ethical Review Committee and was performed under project licenses (80/2304 and 70/7888) granted by the UK Home Office under the Animals (Scientific Procedures) Act 1986. The University of Cambridge is a designated establishment for breeding and scientific procedures under the Animals (Scientific Procedures) Act 1986. Animal experiments performed in Germany were approved by the local administrative institution (Thüringer Landesamt für Verbraucherschutz, Bad Langensalza, Germany).

### Cell transfection

In DNA transfections, cells were transfected using Effectene Transfection Reagent (Qiagen) or Lipofectamine 3000 (Thermo Fisher Scientific) following the manufacturer’s protocol, and typically incubated with transfection reagents for 24 h. For siRNA transfection, cells were transfected with the relevant siRNAs, using Oligofectamine transfection reagent (Invitrogen), according to a protocol modified from Motley et al. (2003). In brief, cells were plated into one well of a six-well plate and transfected after 24 h. Cells were harvested 48–96 h later. Where cells were additionally transfected with a DNA construct during knockdown, DNA transfection was performed 24 h before cells were harvested. The efficiency of siRNA knockdown was verified by immunoblotting cell lysates or by immunofluorescent microscopy of fixed cells, with an antibody against the relevant protein. The following siRNA sequences and concentrations were used: spastin (10 nM): siRNA1, 5′-GAACUUCAACCUUCUAUAA-3′ (Dharmacon D-014070-01); siRNA3, 5′-UAUAAGUGCUGCAAGUUUA-3′ (Dharmacon D-014070-03); IST1 (10 nM): siRNA1, 5′-CCAAGUAUAGCAAGGAAUA-3′ (Dharmacon D-020977-01); strumpellin/KIAA0196/SPG8 (predesigned oligonucleotides used at 10 nM and ordered from Sigma-Aldrich): siRNA1, 5′-CAACAAACGCCUUCGUCAA[dT][dT]-3′ (SASI_Hs02_00346406); siRNA2, 5′-CUAACAGACUCUCGGUACA[dT][dT]-3′ (SASI_Hs01_00210976); siRNA3, 5′-CAAAUGAUCAGAACCAUUA[dT][dT]-3′ (SASI_Hs02_00346407); and siRNA4, 5′-CCACUAUCAGGACCCUUCA[dT][dT]-3′ (SASI_Hs01_00210979).

### IPSC derivation and neural differentiation

Fibroblasts from SPG4 patients and controls were reprogrammed using retroviral transduction of the transcription factors Klf4, c-Myc, Oct4, and Sox2 as previously described (Takahashi et al., 2007; Havlicek et al., 2014; Pérez-Brangulí et al., 2014; Mishra et al., 2016). In brief, fibroblasts seeded in a six-well plate (Corning) in IMDM/Glutamax and 15% FBS (both Invitrogen) were infected three times during 48 h with supernatants of Sox2-, Klf4-, and c-Myc–containing retrovirus and three times the amount of Oct3/4 by spinfection (800 *g* for 60 min; 8 µg/µl Polybrene). 1 d after infection, fibroblasts were detached using TrypLE Express and cultured with hESC medium: DMEM/F12/Glutamax, 20% Knockout Serum Replacement, 1× NEAA (all Invitrogen), 55 µM β-mercaptoethanol (Sigma-Aldrich), 20 ng/ml fibroblast growth factor 2 (FGF2; PeproTech), and 10 µM SB431542 (Sigma-Aldrich) on a feeder layer of irradiated mouse embryonic fibroblasts (EMD Millipore). Colonies that started to appear at ∼12 d after infection were manually isolated and transferred to feeder-free conditions on 24-well plates (Corning) coated with 0.5 mg Matrigel (BD) in mTeSR1 medium (STEMCELL Technologies) and expanded clonally. The iPSC lines maintained a normal karyotype determined by G-banding analysis. Pluripotency was characterized by confirming expression of Nanog and Tra-1-60, and the presence of all three embryonic germ layers after undirected in vitro differentiation, as described previously (Havlicek et al., 2014). To generate NPCs, the iPSCs were transferred to ultra-low-attachment plates (Corning) in mTeSR1 (STEMCELL Technologies) to allow the formation of embryoid bodies (EBs). 24 h later, the medium was changed to DMEM/F12/Glutamax supplemented with N2 and B27 (without VitA) and 1× penicillin/streptomycin (all Invitrogen). After 7 d, the EBs were plated onto plates coated with polyornithine/laminin (Invitrogen). Visible rosettes formed within 1 wk and were manually picked under a stereomicroscope (Olympus). The neural rosettes were dissociated using TrypLE Express (Invitrogen) to form proliferative NPC lines. NPCs were maintained at high density, grown on polyornithine/laminin-coated plates in N2/B27 medium supplemented with 20 ng/ml FGF2 (R&D Systems) and split ∼1:3 every 5–10 d with TrypLE Express (Invitrogen). Terminal NPC differentiation was initiated in N2/B27 medium supplemented with 20 ng/ml brain-derived neurotrophic factor, 20 ng/ml glial cell line–derived neurotrophic factor (both Peprotech), 1 mM dibutyryl-cAMP (AppliChem), and 200 nM ascorbic acid (Sigma-Aldrich) at a density of ∼40,000 cells/cm^2^ on glass coverslips (Thermo Fisher Scientific) coated with polyornithine/laminin. Neurons were cultured under these conditions for 2–4 wk with a half medium change every week (Havlicek et al., 2014; Pérez-Brangulí et al., 2014; Mishra et al., 2016).

### Immunofluorescence microscopy on fixed cells

Cells were fixed at RT in 3.8% (vol/vol) formaldehyde in PBS and permeabilized in PBS containing 0.1% (vol/vol) saponin (Sigma-Aldrich) or 0.1% (vol/vol) Triton X-100 (Sigma-Aldrich). Coverslips were labeled with primary and secondary antibodies as previously described (Connell et al., 2009). In certain cases, soluble cytosolic proteins were removed to allow better imaging of membrane-associated protein, by prefixation treatment with a saponin-based cytosol extraction buffer, as previously described (Edwards et al., 2009). Slides were analyzed with an LSM880 confocal microscope (100× NA 1.40 oil immersion objective, 37°C), LSM710 confocal microscope (100× NA 1.40 oil immersion objective, 37°C), LSM780 confocal microscope (63× NA 1.40 oil immersion objective, 37°C), or AxioImager Z2 Motorized Upright Microscope (63× NA 1.40 oil immersion objective, RT, Axiocam 506; ZEISS), all with ZEN analysis software (ZEISS). Airyscan imaging was performed using an LSM880 microscope fitted with an Airyscan module, using Plan-Apochromat 63×/1.4-NA Oil differential interference contrast (DIC) M27 objective at RT. Images were subsequently processed using ImageJ, Adobe Photoshop, Adobe Illustrator, and ZEN analysis software. 3D image preparation was performed using Imaris (Bitplane). Colocalization of proteins was quantified using Volocity (PerkinElmer).

### Endosomal tubulation counts on fixed cells

Cells were processed for immunofluorescence microscopy and imaged with an AxioImager Motorized Upright Microscope under a 63×/1.4-NA oil immersion objective as described earlier. Tubulation was quantified using one of two methods: (a) as previously (Allison et al., 2013), images of typically 30 cells per condition were randomized and the number of tubules per cell longer than 2 µm, up to a maximum of 20 tubules, was counted blind; and (b) images of typically 30 cells per experimental condition were randomized and scored blind for the presence of tubules longer than 2 µm. The percentage of cells with such tubules was then calculated. This technique was less labor-intensive than (a) but robustly detected tubulation phenotypes.

### ciM6PR trafficking assay

After a 48-h siRNA knockdown of the relevant protein, HeLa cells stably expressing GFP-GOLPH3 and CD8-ciM6PR were subjected to ciM6PR trafficking assays as described in Seaman (2007). In brief, after incubation in medium containing anti-CD8 at 4°C, cells were incubated for 24–30 min at 37°C before fixation and processing for immunofluorescence microscopy with an LSM880 confocal microscope as described earlier. Colocalization of proteins was quantified using Volocity.

### Lysosome quantification

To determine the percentage of cells with large lysosomes, fixed cells labeled with LAMP1 were processed for immunofluorescence microscopy and imaged with an AxioImager Motorized Upright Microscope as described earlier. At least 50 cells were recorded per experimental condition. Images were randomized, and the largest lysosome per cell was measured using ZEN. In axons, the number of lysosomes in an axonal swelling (defined as an axonal region labeling with tau and >2× the diameter of the adjacent axon) and in the same length of adjacent axon was counted in 20 axons per experiment and expressed per micrometer of axon.

To determine the overall distribution of lysosomal size, the terminal degradative compartment was labeled by uptake of fluorescent dextran and imaged under assessment of lysosomal pH. Images were analyzed with ImageJ to identify individual lysosomal puncta above a cutoff threshold of brightness and to measure their surface area. Individual cells were delineated from the bright-field DIC signal, and central lysosomes were defined as those inside a region of interest drawn 5 µm from the edge of the cell.

### Measurement of lysosomal pH

Lysosomal pH measurement was based on previously described methods (Bright et al., 2016; Johnson et al., 2016). In brief, cells were grown to 80% confluence before incubation for 4 h at 37°C in medium containing 1 mg/ml dextran conjugated to Oregon Green (pH sensitive) or tetramethylrhodamine (pH insensitive). This was followed by a 20-h unlabeled chase before imaging with live-cell microscopy at 37°C using an LSM780 microscope (63× NA, 1.40 oil objective). ImageJ was used to delineate individual puncta and determine the ratio of Oregon Green to tetramethylrhodamine signal for each punctum. The pH of each punctum was determined by analysis against a standard curve of fluorescence ratio, generated using cells incubated with 10 µm nigericin and 5 µm monensin in buffers ranging from pH 4.5 to 6.5, for 10 min before imaging.

### Live-cell microscopy of endosomal tubules and ER

For imaging of GFP-SNX1 alone, cells were grown on 25 mmØ coverslips to 60% confluence. Cells were then washed in PBS before imaging in Live Cell Imaging Solution (Thermo Fisher Scientific) supplemented with 10% (vol/vol) FCS. Cells were imaged using a 63×/1.40-NA iil DIC objective (ZEISS) on a 37°C incubated AxioObserver Z1 inverted microscope fitted with a CSU-X1A Spinning Disk scanhead (Yokogawa) and an Andor iXon Ultra 897 EM-CCD (512 × 512) camera driven by ZEN Blue software (ZEISS). Imaging of GFP-SNX1 was performed at 400-ms exposure per frame, continuously for 3 min. Images were run through ZEN Blue’s Single Pixel Filter (threshold 1.5) before export. Images were subsequently processed using ImageJ, Adobe Photoshop, Adobe Illustrator, and ZEN.

For imaging of ER–endosome dynamics of MRC5 cells, cells were grown on 14-mm Microwell No. 1.5 coverglass MatTek dishes to 60% confluence. Cells were then washed in phenol red–free DMEM supplemented with FCS, l-glutamine, and penicillin/streptomycin before being imaged in the same medium. Cells were imaged using a Nikon 100×/1.49-NA Oil objective on a 37°C incubated Nikon Eclipse Ti inverted microscope fitted with a CAIRN MultiCam beamsplitter with two Andor iXON Ultra 897 EM-CCD (512 × 512) cameras driven by Nikon NIS-Elements software. Imaging of GFP-SNX1 and RFP-KDEL was performed at 400-ms exposure per frame simultaneously in both colors, continuously for 3 min.

In the processing of GFP-SNX1 figures, a mild Gaussian blur function was applied to help remove hot pixels. In the processing of ER–endosome figures, images were deconvolved using Huygens Professional software.

### Live-cell image quantification

For quantification of GFP-SNX1 endosomal tubule dynamics, tubules (defined as linear structures having a length of >1 µm) were observed in five cells per condition per experimental repeat. Parameters quantified for each tubule were maximum tubule length, tubule duration from formation to fission or collapse, and fate (fission at base, fission elsewhere on tubule, or collapse into parent endosome). Fission at the tubule base was defined as fission occurring within 1.5 µm of the endosome body. In quantification of GFP-SNX1 endosomal tubules in relation to RFP-KDEL labeled ER, GFP-SNX1 tubules >1 µm long were identified within regions of the cell with resolvable ER tubules, and five cells were observed per condition per experimental repeat. Parameters quantified were whether endosomal tubule fission occurred in relation to an ER tubule and the total duration an endosomal tubule contacted an ER tubule between its formation until fission or collapse. All quantifications were performed blind by randomization of datasets. The total number of tubules analyzed in each set of experiments was as follows: MRC5 (spastin KD experiments)/GFP-SNX1 tubule duration analysis, *n* = 6, in total 450 tubules analyzed in mock conditions, 450 in knockdown conditions; MRC5 (spastin KD experiments)/GFP-SNX1 tubule fate analysis, *n* = 5 experiments, in total 375 tubules analyzed in mock conditions, 375 in knockdown conditions; MRC5 (IST1 KD experiments)/GFP-SNX1 tubule duration and fate analysis, *n* = 6 experiments, in total 450 tubules analyzed in mock conditions, 450 in knockdown conditions; Spastin^N384K^ MEFs/GFP-SNX1 tubule duration and fate analysis, *n* = 4 experiments, in total 300 tubules analyzed for each genotype; MRC5 (spastin KD experiments)/GFP-SNX1 with resolvable RFP-KDEL ER tubules analysis, *n* = 3, in total 211 tubules analyzed in mock conditions, 289 in knockdown conditions.

### Cathepsin D secretion assay

After a 48-h siRNA knockdown of the relevant protein, HeLa cells were processed for cathepsin D secretion as described in Hao et al. (2013). In brief, cells were incubated for 16 h in OPTI-MEM, then protein was precipitated from culture medium with TCA, suspended in SDS-sample buffer, and immunoblotted. Equal loading was determined by Coomassie staining of proteins precipitated from the medium and immunoblotting of adherent cell lysates. Immunoblot band intensity was quantified in ImageJ.

### Electron microscopy

Fibroblasts and murine neurons were grown on glass or Thermanox (Thermo Fisher Scientific) plastic coverslips and fixed with 2% PFA, 2.5% glutaraldehyde, and 0.1 M cacodylate buffer (pH 7.2). Human iPSC-derived neurons were grown on glass or plastic coverslips (Invitrogen) and fixed with 2.5% glutaraldehyde and 0.1 M sodium phosphate buffer (pH 7.2). HeLa cells were processed as pellets for experiments requiring membrane contact site quantitation. HeLa cells were grown on plastic dishes and fixed with 2% PFA, 2.5% glutaraldehyde, and 0.1 M cacodylate buffer, pH 7.2, before being scraped and spun into a pellet. All samples were postfixed with 1% osmium tetroxide:1.5% potassium ferricyanide before being incubated with 1% tannic acid to enhance contrast. Cells were dehydrated using increasing percentages of ethanol before being embedded onto EPON stubs or beam capsules. Resin was cured overnight at 65°C, and coverslips were removed using a heat-block (plastic) or repetitive freeze-thaw cycles with liquid nitrogen (glass). Ultrathin (50- to 70-nm) conventional sections were cut using a diamond knife mounted to a Reichart ultracut S ultramicrotome. Sections were collected onto copper grids stained using lead citrate. Sections were viewed on a FEI Tecnai transmission electron microscope at a working voltage of 80 kV.

The total number of organelles analyzed in EM experiments to analyze ER–endosome contacts was as follows: HeLa cells (spastin KD), *n* = 3 biological repeats; early endosomes, mock = 23, KD = 21; multivesicular bodies, mock = 50, KD = 68; Spastin^N384K^ MEFs, early endosomes, WT = 36, spastin^wt/N384K^ (HET) = 27, spastin^N384K/N384K^ (KI) = 28; multivesicular bodies, WT = 25, Het = 27, KI = 29.

### BSA-gold labeling

Gold-conjugated BSA was prepared as previously described (Slot and Geuze, 1985). Cells were grown on Thermanox plastic coverslips, and 10 nm BSA-gold was incubated continuously with cells for 2 h before fixation.

### Membrane contact site EM quantitation

Cells were processed as a pellet for conventional EM to ensure random orientation of cells. Contact sites were classified as regions of membrane apposition within 20 nm (Eden et al., 2010). Early endosomes were defined as vacuolar organelles with one or no intraluminal vesicles. MVBs were defined by the presence of ≥2 intraluminal vesicles but no other intraluminal membrane content. ER was distinguished from endosomal tubules by its reticular structure and by the presence of ribosomes. Occasional endosomal tubules could be seen emanating from endosomes, and these were distinguishable from ER, being less reticular with a more lucent lumen.

### Quantification of endolysosomal ultrastructural morphology

In each experimental condition, randomly chosen sections were analyzed until the morphology of 50 multivesicular bodies (defined as containing intraluminal vesicles) or lysosomal organelles was quantified. The appearances were classified into three groups based on the predominant phenotype for each organelle. Vacuolar structures were enlarged structures that were electron lucent and contained only sparse linear membrane or a few intraluminal vesicles; dense network organelles contained material that often had a honeycomb appearance or had dense coiled or stacked whorls of membrane; and organelles with membrane cords had thick electron-dense belt or cordlike linear structures.

### Statistical analysis

Differences from ≥3 independent experiments examining (a) the percentage of cells with tubules >2 µm, (b) the number of tubules per cell, (c) the percentage of cells with largest lysosome >1.8 or 2 µm, (d) Pearson’s coefficient between colocalizing proteins, (e) number of lysosomes per μm of axon or per cell, (f) endolysosomal ultrastructural morphology, or (g) quantification of bands on immunoblot by densitometry were compared by paired two-tailed Student’s *t* tests. All live-cell data parameters in MRC5 cells were compared by paired two-tailed Student’s *t* tests. Live-cell results in MEFs were compared using analysis of variance (ANOVA) for the effect of genotype. Differences in ER–endosome contacts detected by EM were analyzed by paired two-tailed *t* tests in HeLa cells and by ANOVA, for the effect of genotype, in MEFs. Unpaired two-tailed Student’s *t* tests were used in experiments with fluorescent dextran to quantify lysosomal size, number, and position. Differences between the mean pH of puncta in wild-type versus spastin-depleted cells were analyzed using a two-tailed Mann–Whitney *U* test. Statistical analysis was performed using GraphPad Prism 5.01 for Windows or JMP 10 statistical software (SAS Institute).

### Online supplemental material

Fig. S1 contains supporting data to show that spastin promotes endosomal tubule fission at ER contacts. Fig. S2 contains supporting data to show that IST1–spastin interaction regulates endosomal tubule fission. Fig. S3 contains data supporting a role for spastin in efficient endosome to Golgi traffic. Fig. S4 contains supporting data demonstrating that spastin regulates lysosomal enzyme traffic and lysosomal morphology. Fig. S5 contains supporting data showing siRNA rescue experiments that characterize spastin and IST1 sequences required to regulate lysosomal size. Video 1 shows endosome dynamics in wild-type and spastin-depleted MRC5 cells stably expressing GFP-SNX1. Video 2 shows endosome dynamics in MEFs from spastin mouse model, stably expressing GFP-SNX1. Video 3 shows that endosomal tubule fission occurs at ER tubules in mock-transfected and spastin-depleted MRC5 cell. Video 4 shows endosomal tubule fission dynamics in a wild-type MRC5 cell and a cell depleted of IST1. Video 5 shows that spastin and IST1 localize at endosomal tubule constrictions; 3D reconstruction of a *z*-stack of a single SNX1 tubule.

## Supplementary Material

Supplemental Materials (PDF)

Video 1

Video 2

Video 3

Video 4

Video 5
