# Supplementary material for: Defects in ER–endosome contacts impact lysosome function in hereditary spastic paraplegia
Source: J Cell Biol. 2017 May 1;216(5):1337–55. doi: 10.1083/jcb.201609033 (PMC5412567; doi:10.1083/jcb.201609033)
Supplement: Supplemental Materials (PDF) [file JCB_201609033_sm.pdf]

Allison et al., https://doi.org/10.1083/jcb.201609033

THE JOURNAL OF CELL BIOLOGY

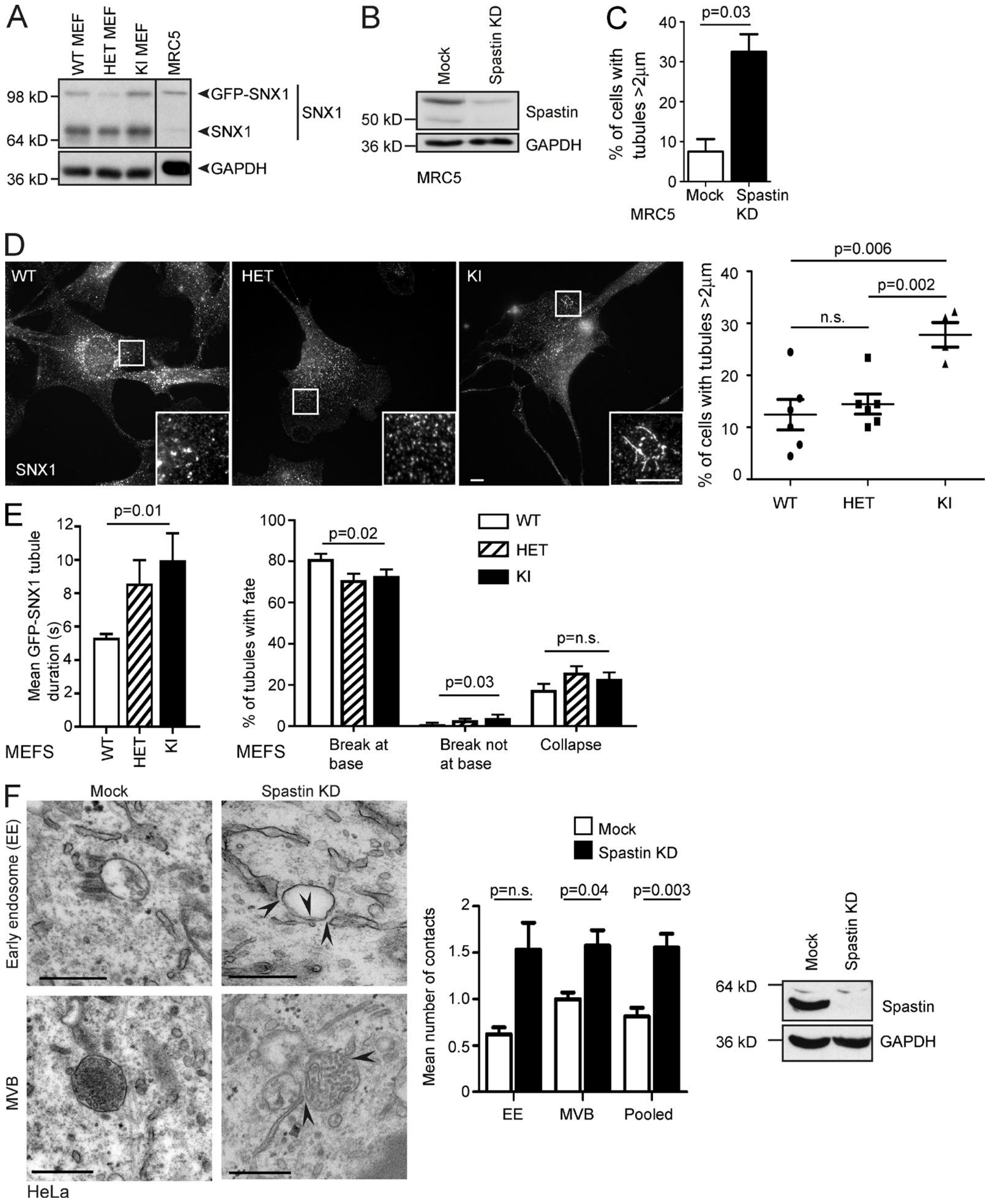

Figure S1. **Spastin promotes endosomal tubule fission at ER contacts.** (A) GFP-SNX1 expression levels in cell lines used for live cell imaging. Cell lines stably expressing GFP-SNX1 were immunoblotted against SNX1 (top). In this and subsequent immunoblots, GAPDH signal (bottom) is shown to verify equal lane loading. Bands representing SNX1 or GFP-SNX1 are indicated. MEFs were derived from animals with the following genotypes: WT, spastin$^{wt/wt}$; HET, spastin$^{wt/N384K}$; KI, spastin$^{N384K/N384K}$. (B) Immunoblot probed with the antibodies indicated, illustrating typical spastin siRNA KD in MRC5 cells. (C) MRC5 cells were mock-transfected or subjected to spastin siRNA KD, fixed, and visualized by confocal immunofluorescence microscopy (IF) for endogenous SNX1. The mean percentage of cells containing at least one SNX1 tubule longer than 2 µm was quantified (*n* = 4 biological repeats, 30 cells per condition counted in each repeat). (D) MEFs from spastin^{N384K} mice with the genotypes shown were fixed and visualized by IF for endogenous SNX1. The percentage of cells containing at least one SNX1 tubule longer than 2 µm was quantified and plotted in the corresponding scatterplot. Each data point represents an individual animal; 30 cells analyzed per animal. (E) MEFs stably expressing GFP-SNX1, and derived from spastin^{N384K} mice with the genotypes shown, were visualized by live-cell microscopy. The mean duration (from formation to fission or collapse) of GFP-SNX1 tubules is plotted in the left histogram, and the fate of each GFP-SNX1 tubule is plotted in the histogram on the right (*n* = 4 biological repeats; see Materials and methods for the number of tubules analyzed in this and other live-cell experiments). (F) HeLa cells were mock-transfected or subjected to spastin KD. Cells were processed for EM, and contacts between endosomal structures and ER tubules were analyzed. Arrows indicate points of ER contact. The mean number of contacts with each class of structure is quantified in the corresponding histogram (pooled, EE and MVB results combined). *n* = 3 biological repeats; see Materials and methods for number of endosomal structures analyzed. In F, representative immunoblots with the antibodies indicated illustrate typical spastin KD efficiency obtained in experiments with HeLa cells. All histograms and plots show mean ± SEM. Bars: (EM) 500 nm; (IF) 10 µm. P-values generated by two-tailed Student's *t* test, except E, in which ANOVA was used to analyze the effect of genotype.

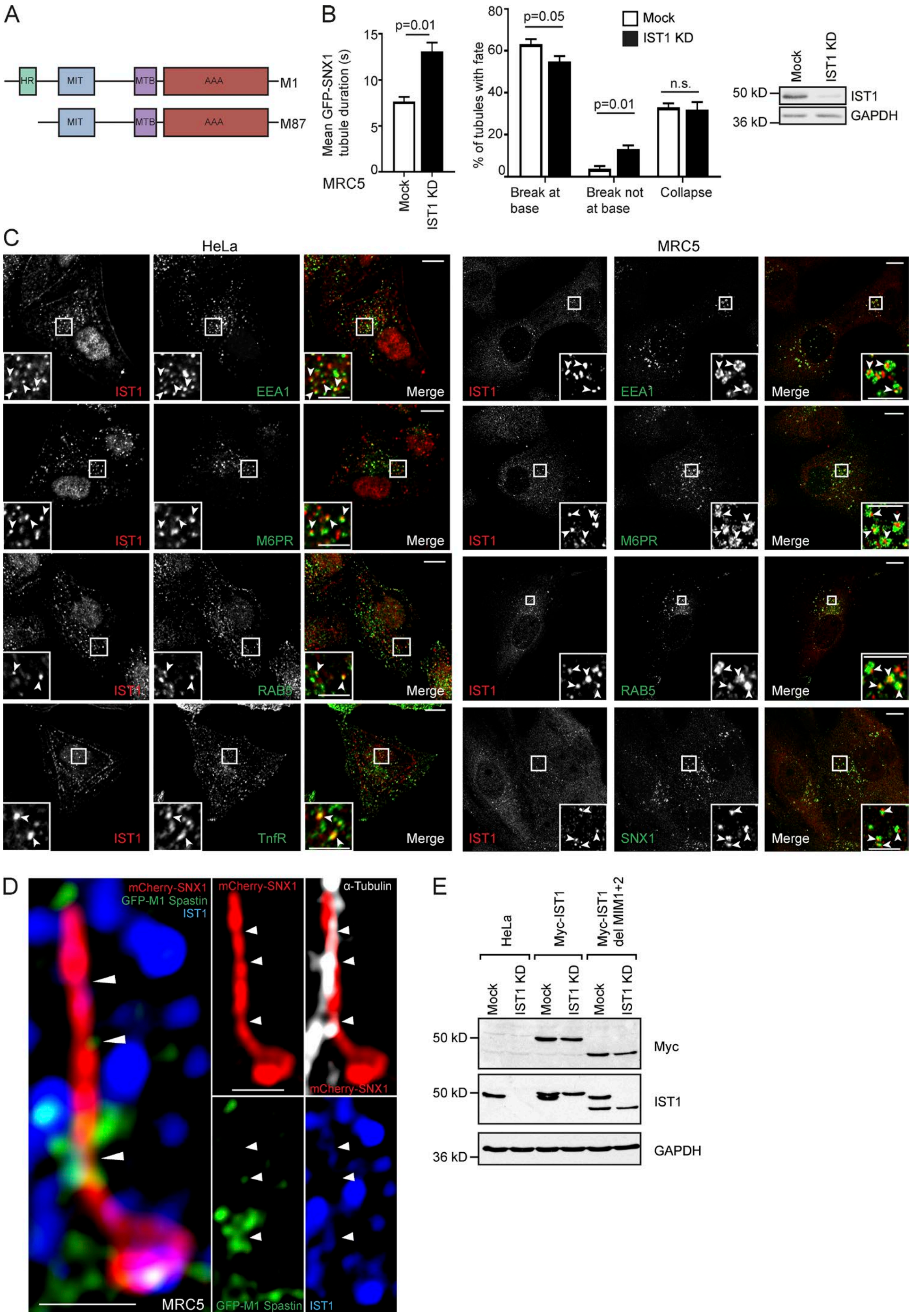

Figure S2. **IST1 interacts with spastin to regulate endosomal tubule fission.** (A) Schematic diagram of M1- and M87-spastin isoforms. HR, hydrophobic region; MIT, MIT domain; MTB, microtubule binding domain; AAA, AAA ATPase domain. (B) MRC5 cells stably expressing GFP-SNX1 were mock-transfected or subjected to IST1 KD, then visualized by live cell microscopy. The duration of each GFP-SNX1 tubule, from formation to fission or collapse, was measured, and the mean duration was plotted in the left panel. The fate of each GFP-SNX1 tubule is plotted in the right panel (n = 6 biological repeats). The representative immunoblot shows typical IST1 KD efficiency. (C) HeLa and MRC5 cells were fixed and visualized by confocal immunofluorescence microscopy (IF) using antibodies to the proteins shown. Magnified images of the boxed areas are shown in the insets. Colocalized or (in the case of IST1/SNX1 labeling in MRC5 cells) juxtaposed signal is indicated with arrowheads. Experiments in HeLa cells used prefixation treatment with a cytosol extraction buffer, to remove obscuring soluble cytosolic IST1 and allow visualization of membrane-associated signal. This was not required in MRC5 cells, in which cytosolic IST1 signal was less prominent. Bars: (main panels) 10 µm; (magnified insets) 5 µm. (D) MRC5 cells stably expressing GFP-M1-spastin and mCherry-SNX1 were fixed and labeled with anti-IST1 and anti–α-tubulin antibodies, then visualized with four-color Airyscan IF. The images shown are of a single *z*-slice. The small panels show selected channels from the large image, or in the case of the top right panel, SNX1 and α-tubulin (for clarity, tubulin labeling was excluded from the large image). Note that several SNX1 tubule constrictions are visible (examples indicated by arrowheads). IST1 and spastin are closely apposed at a constriction close to the base of the SNX1 tubule (lowest arrowhead), whereas spastin or IST1 signal is present at other constriction points along the tubule (upper two arrowheads). See Video 5 for a 3D reconstruction of a *z*-series that incorporates this image. Bars, 1 µm. (E) Immunoblots with the antibodies indicated, to verify protein expression levels for the rescue experiments shown in Fig. 2 C. All histograms show mean ± SEM. P-values generated by paired two-tailed Student's *t* test.

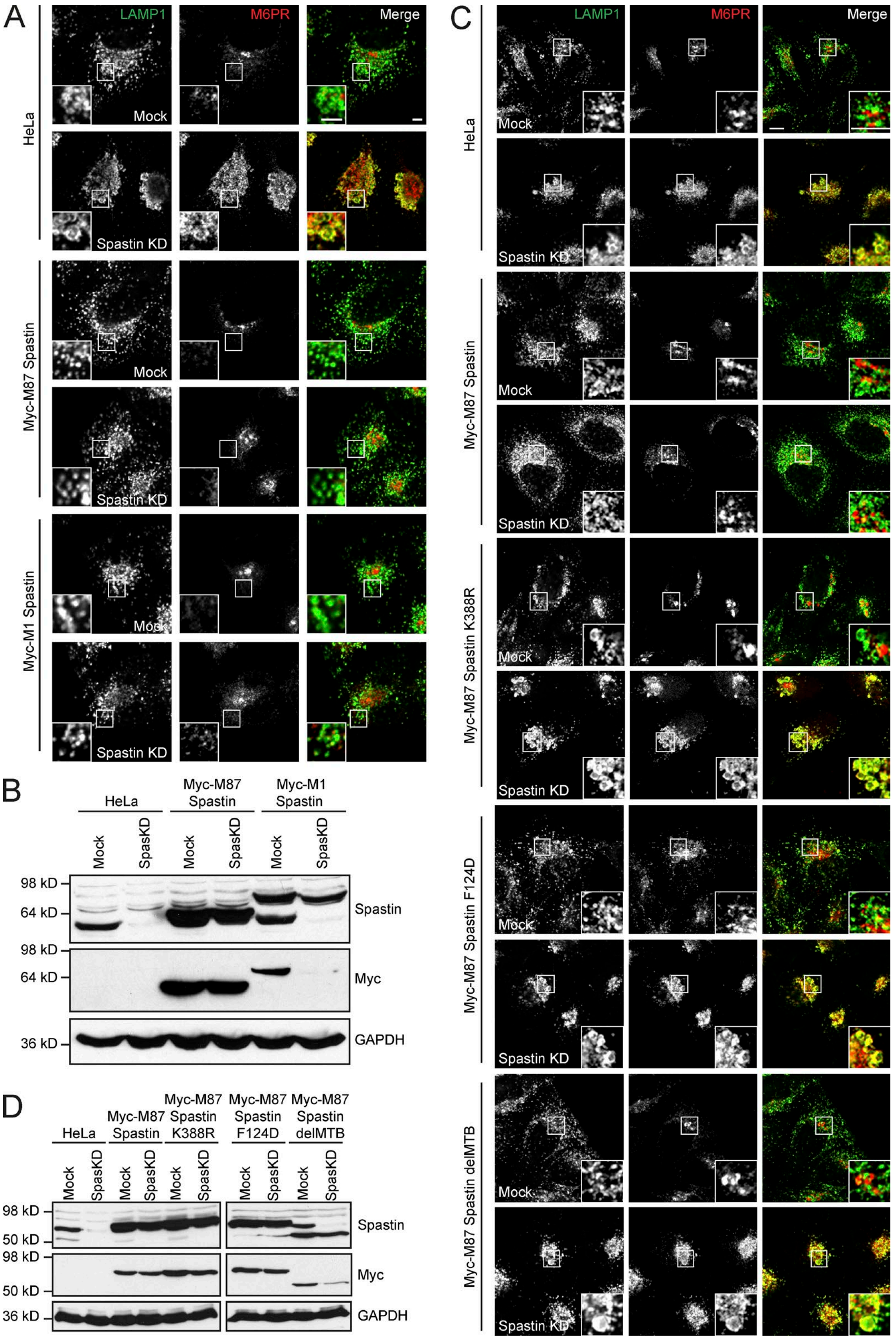

Figure S3. **Spastin is required for efficient endosome-to-Golgi traffic.** (A and C) Representative micrographs corresponding to the results shown in Fig. 3 (B and C). Wild-type HeLa cells or HeLa cells stably expressing the siRNA-resistant spastin proteins indicated were subjected to mock-transfection or KD of endogenous spastin, processed for confocal immunofluorescence microscopy (IF), and labeled with antibodies to M6PR and LAMP1. Insets show higher-magnification views of the boxed areas. (B and D) Corresponding immunoblots for A and C to verify expression levels of endogenous and exogenous proteins. Bars, 10 µm.

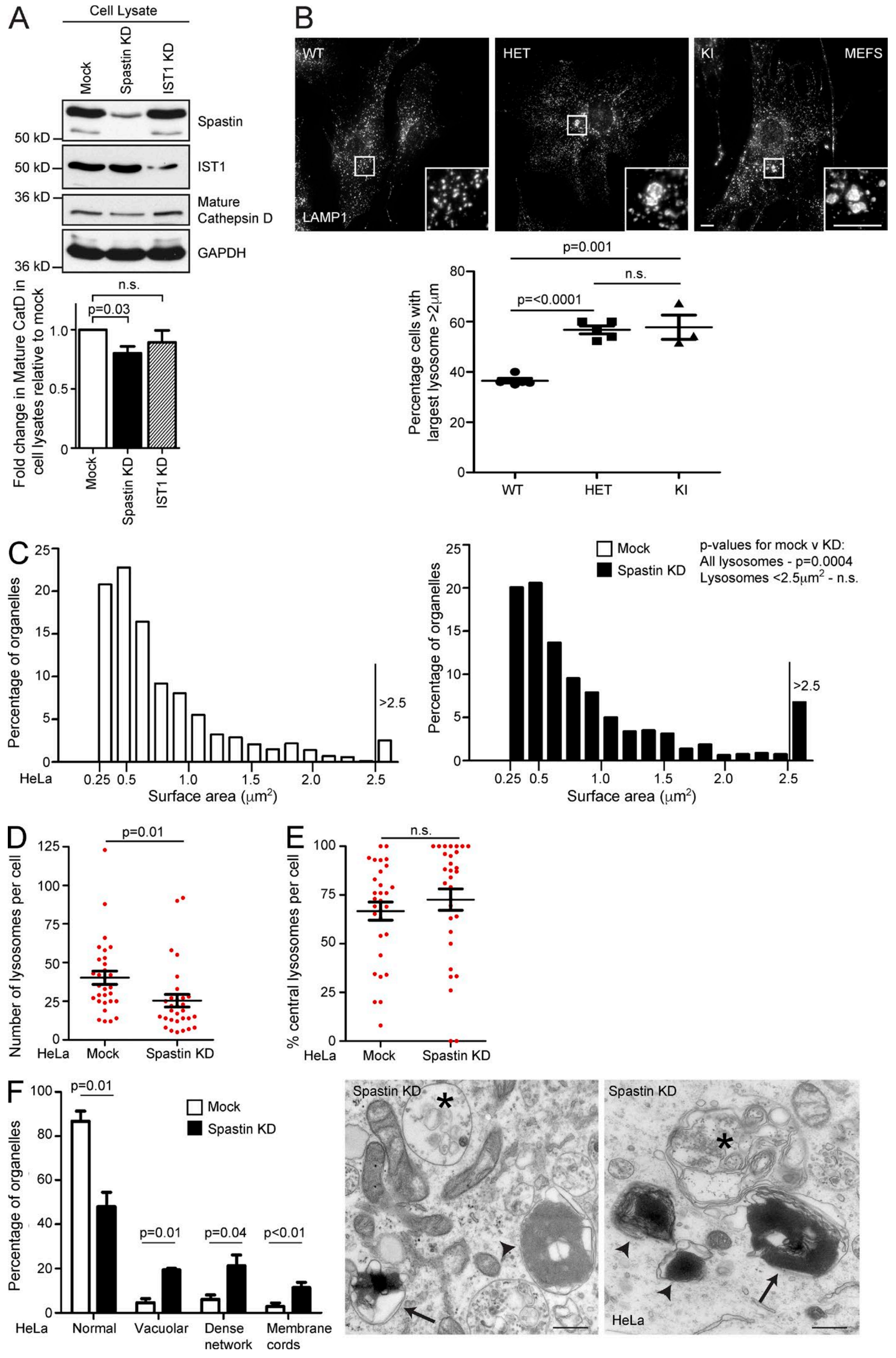

Figure S4. **Spastin regulates lysosomal enzyme traffic and lysosomal morphology.** (A) Immunoblotting of cell lysates corresponding to the media samples shown in Fig. 4 A, to verify spastin and IST1 depletion and show cellular cathepsin D expression levels. Quantification of cathepsin D immunoblot band density is shown in the corresponding histogram (*n* = 5). (B) MEFs derived from spastin^{N384K} mice with the genotypes shown were fixed and processed for confocal immunofluorescence microscopy (IF) against endogenous LAMP1. The diameter of the largest lysosome in each cell was measured, and the percentage of cells with the largest lysosome >2 µm in diameter is shown in the corresponding plot. Each data point on the plot represents the mean percentage in 300 cells derived from an animal. (C–E) Characterization of lysosomes in mock-transfected or spastin-depleted cells. Lysosomes were visualized by live-cell imaging of fluorescent dextran in the terminal degradative compartment (achieved by a 4-h pulse followed by 20-h chase). Lysosome number and size were then analyzed. The size distribution is shown in C (*n* = 869 lysosomes mock; 796 in spastin-depleted cells), and the mean number of lysosomes per cell is shown in D. The proportion of central lysosomes in each cell (i.e., those that did not reside within 5 µm of the cell edge) is shown in E. (F) Quantification of lysosomal ultrastructural morphology in mock-transfected cells or cells knocked down for spastin by siRNA transfection. Categorization of organelles is defined in Materials and methods. The leftmost corresponding image shows typical organelles with a vacuolar appearance (*), a dense network of membrane (arrowhead), or containing thick membrane cords (arrow). (right) An organelle with a relatively vacuolar appearance (*) along with three other organelles with a dense network of membrane (arrow and arrowheads). Note that even smaller organelles could have abnormal appearances (arrowheads). For quantitation purposes, the predominant phenotype was scored in 50 randomly chosen organelles (either multivesicular bodies with intraluminal vesicles or lysosomes) per experimental condition. The percentage of organelles with each morphology was plotted from *n* = 3 biological experimental repeats. Bars: (EM) 500 nm; (IF) 10 µm. All histograms and plots show mean ± SEM. P-values generated by two-tailed Student's *t* test.

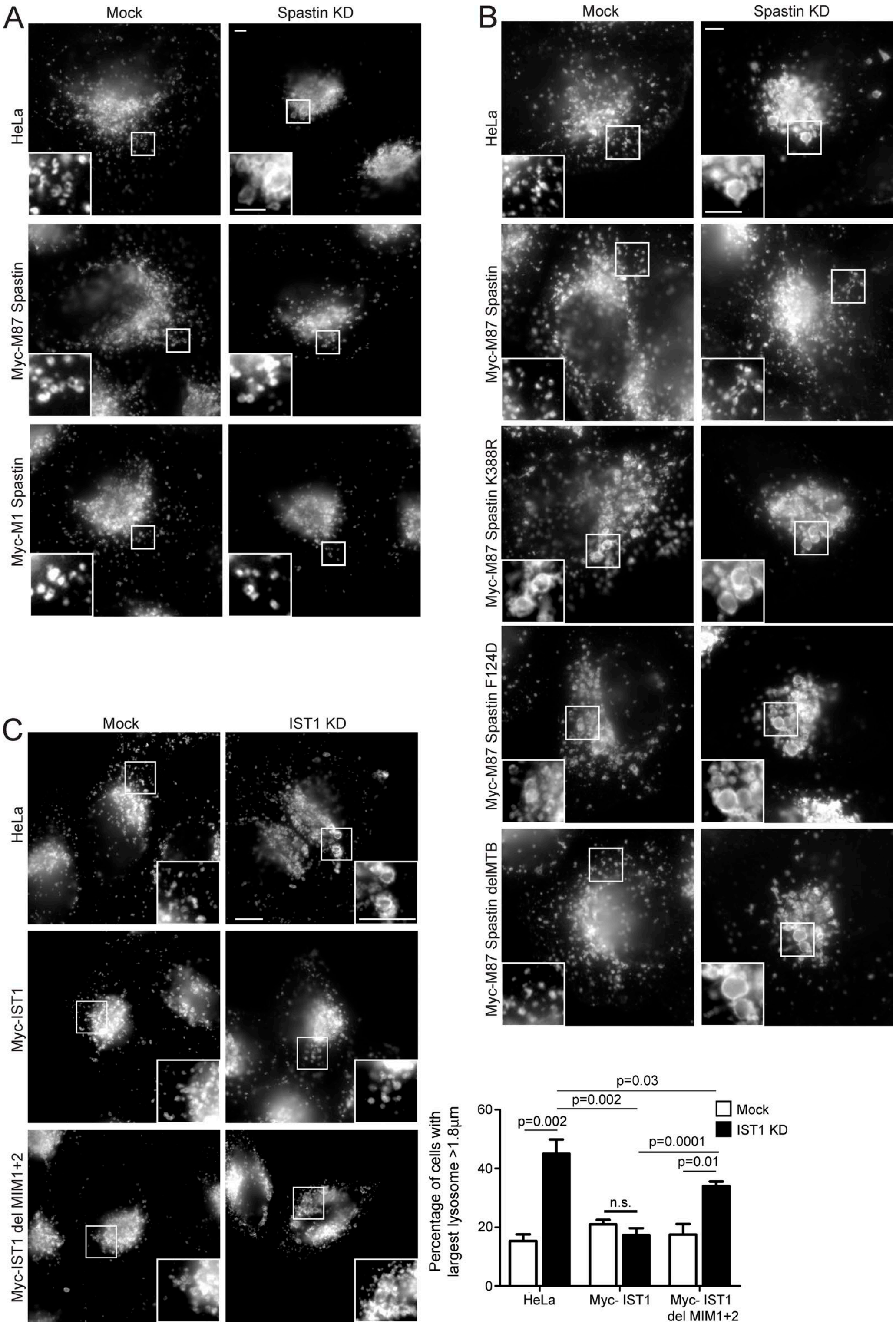

Figure S5. **Spastin and IST1 sequences required to regulate lysosomal size.** (A and B) Representative micrographs corresponding to the histograms shown in Fig. 4 (F and G), respectively. Wild-type HeLa cells or HeLa cells stably expressing the siRNA-resistant spastin proteins indicated were subjected to mock-transfection or KD of endogenous spastin, fixed, and processed for confocal immunofluorescence microscopy (IF) to visualize endogenous LAMP1. The size of the largest lysosome was measured in 100 cells per condition in each experiment, and the mean percentage of cells with largest lysosome >1.8 µm is shown in Fig. 4. Corresponding immunoblots are shown in Fig. S3. (C) Wild-type HeLa cells, or HeLa cells stably expressing the siRNA-resistant proteins indicated, were mock-transfected or subjected to endogenous IST1 KD. Cells were fixed and visualized by IF for LAMP1, and the diameter of the largest lysosome in each cell was measured. The percentage of cells with the largest lysosome >1.8 µm is plotted in the corresponding histogram (*n* = 6 experiments; 100 cells counted per condition in each experiment). Representative corresponding immunoblots are shown in Fig. S2 E. Histogram shows mean ± SEM. Bars, 10 µm. P-values generated by two-tailed Student's *t* test.

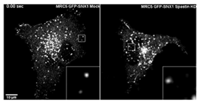

Video 1. **Endosome dynamics in wild-type and spastin-depleted MRC5 cells stably expressing GFP-SNX1.** Representative example of a mock-transfected (left) or spastin siRNA-transfected (right) MRC5 cell stably expressing GFP-SNX1. Insets show a higher-magnification view of endosomal tubule fission events indicated by boxes in the main movies and correspond to panels in Fig. 1 A. Note that the GFP-SNX1 tubules are much longer in the cell depleted of spastin, and that although fission events do occur, the length of time taken between tubule formation and fission is longer than in the mock-transfected cell. Tubule collapse events are also seen. These and subsequent videos are excerpts from 3-min videos taken at 400 ms/frame and are displayed at 15 frames/s.

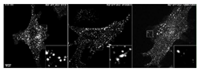

Video 2. **Endosome dynamics in MEFs stably expressing GFP-SNX1.** Representative examples of MEFs stably expressing GFP-SNX1, derived from Spastin$^{wt/wt}$ (left), Spastin$^{wt/N384K}$ (center), or Spastin$^{N384K/N384K}$ (right) animals. Note the development of elongated GFP-SNX1 tubules in the Spastin$^{N384K/N384K}$ MEFs that are slow to break from the parent endosome. Insets show a higher-magnification view of endosomal tubule fission events indicated by boxes in the main videos.

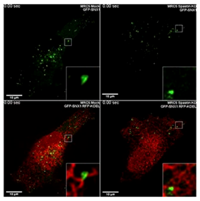

Video 3. **Endosomal tubule fission occurs at ER tubules.** Representative examples of MRC5 cells stably expressing GFP-SNX1 and transiently expressing RFP-KDEL. Left, a mock siRNA-transfected cell; right, a spastin siRNA-transfected cell. Top, only the GFP-SNX1 tubule fission events; bottom, the GFP-SNX1 tubule with RFP-KDEL. Insets show a higher-magnification view of the endosomal tubule fission events shown in the boxed region in the larger panels and correspond to Fig. 1 B. Note that SNX1 tubule fission occurs at or very close to where it crosses an ER tubule and appears to be preceded by constriction of the SNX1 tubule. The spastin siRNA-transfected cell shows a longer duration of endosome–ER contact before fission. Videos were imaged in both channels simultaneously at 400 ms per frame for 3 min and are shown at 15 frames/s.

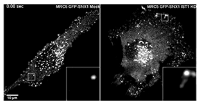

Video 4. **Endosomal tubule fission dynamics in MRC5 cells depleted of IST1.** Representative examples of mock siRNA-transfected (left) or IST1 siRNA-transfected (right) MRC5 cells stably expressing GFP-SNX1. Insets show a higher-magnification view of the endosomal tubule fission events highlighted by the boxes in the main images. Note the striking similarity to cells lacking spastin, with the presence of long GFP-SNX1 tubules that are slow to break from the parent endosome.

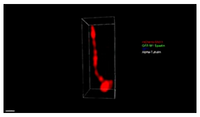

Video 5. **Spastin and IST1 localize at endosomal tubule constrictions.** MRC5 cells stably expressing GFP-M1-spastin and mCherry-SNX1 were fixed and labeled with anti-IST1 and anti–α-tubulin antibodies, then visualized with four-color Airyscan superresolution confocal immunofluorescence microscopy. The movie is a 3D reconstruction of a *z*-stack of a single SNX1 tubule (a single *z*-slice from this stack is shown in Fig. S2 D). Note that several tubule constrictions are visible. IST1 and spastin are closely apposed at constrictions close to the base of the SNX1 tubule, and the entire structure is closely related to microtubule labeling. 3D rendering was performed with Imaris software.
